# Outcome-specific value of longitudinal clinical information and model complexity for dynamic prediction of type 2 diabetes-related outcomes: a pooled landmarking study

**DOI:** 10.3389/fendo.2026.1964592

**Published:** 2026-10-02

**Authors:** Meiling Fu, Xiaohong Liu, Ting Shi, Yuxuan Lu, Jie Zou, Xiaowei Huang, Qingmei Pan, Yun Chen, Yinqin Zhong

**Affiliations:** 1 Shenzhen Hospital (Futian), Guangzhou University of Chinese Medicine, Guangdong, Shenzhen, China; 2 The Sixth Clinical Medical College, Guangzhou University of Chinese Medicine, Guangdong, Shenzhen, China

**Keywords:** dynamic prediction, internal validation, landmark analysis, longitudinal information, machine learning, type 2 diabetes

## Abstract

**Objective:**

To evaluate the outcome-specific incremental value of longitudinal clinical history and model complexity for annual dynamic prediction of multiple type 2 diabetes-related outcomes using pooled landmarking.

**Methods:**

We included 646 patients with complete annual records from baseline through year 3. Landmark times at years 1 and 2 were used to predict the first recorded occurrence of hypertension, coronary heart disease, diabetic kidney disease, diabetic retinopathy, or metabolic dysfunction-associated steatotic liver disease during the subsequent year. Four progressively expanded feature sets were compared using elastic-net logistic regression and Light Gradient Boosting Machine. The primary history-current estimand was a paired within-algorithm contrast with the risk set, validation splits, preprocessing, tuning, calibration, and evaluation records held constant. Internal validation used patient-level repeated nested cross-validation. We also evaluated fully nested model selection and model-development stability using 500 patient-level bootstrap replicates with out-of-bag evaluation.

**Results:**

Pooled out-of-fold area under the receiver operating characteristic curve ranged from 0.722 to 0.952 across the five representative models. The paired history-current comparison showed higher AUC for hypertension with elastic-net (ΔAUC 0.097, 95% confidence interval 0.057 to 0.136) and LightGBM (0.118, 0.081 to 0.158), and for metabolic dysfunction-associated steatotic liver disease with elastic-net (0.022, 0.002 to 0.042) and LightGBM (0.025, 0.009 to 0.041). The other three outcomes showed no comparable improvement. Algorithm differences were modest and outcome dependent.

**Conclusion:**

In this single-center cohort with complete annual T0–T3 follow-up, the incremental value of longitudinal information and model complexity was outcome specific. Longitudinal history showed greater benefit for selected outcomes, whereas algorithm differences were modest and outcome dependent. The smaller MASLD signal was supported primarily by the pooled L1–L2 analysis. Independent external validation is required before clinical application.

## 1 Introduction

Type 2 diabetes is managed over years rather than at a single visit. Glycemic control, body weight, blood pressure, kidney function, and other metabolic measures may change during follow-up, together with the risk of cardiovascular, renal, retinal, and hepatic complications. Current guidelines emphasize repeated risk assessment and adjustment of management as new clinical information becomes available (1–9). Risk estimates that are updated after each clinical review may therefore be more useful for routine care than predictions based only on baseline information.

Against this background, regression and machine-learning methods are increasingly used to predict diabetes-related outcomes (10–16), including hypertension (HTN), coronary heart disease (CHD), diabetic kidney disease (DKD), diabetic retinopathy (DR), and metabolic dysfunction-associated steatotic liver disease (MASLD) (17–21). Most published models, however, address a single outcome or start prediction from a fixed baseline. In longitudinal data, baseline values, current measurements, and historical changes are also often entered together, which makes the contribution of prior history difficult to separate from that of the most recent visit. More complex machine-learning algorithms have not consistently outperformed simpler statistical approaches across diabetes-related prediction tasks (22). This leaves two practical questions for repeated risk assessment: how much longitudinal information should be retained, and when does additional model complexity add value?

One way to separate these contributions is landmarking. At prespecified follow-up times, it resets the prediction origin and uses only information available up to that point to predict events over a fixed subsequent window (23–26). For a given outcome, patients with a recorded event before the landmark leave that outcome-specific risk set. Pooled landmarking combines records from several landmark times while preserving temporal order and patient identity, allowing information depth and modeling approach to be compared within the same repeated-risk framework.

On this basis, we used annual follow-up data from a baseline visit (T0) and three annual follow-up visits (T1-T3) to construct one-year prediction tasks at landmark time 1 (L1, corresponding to T1) and landmark time 2 (L2, corresponding to T2) for five type 2 diabetes-related outcomes. Four progressively expanded information sets were evaluated with elastic-net logistic regression and Light Gradient Boosting Machine (LightGBM). The primary history-current estimand was defined separately within elastic-net and LightGBM, with the outcome-specific risk set, outer validation splits, preprocessing, tuning framework, calibration procedure, and evaluation records held constant. Reporting was guided by the Transparent Reporting of a multivariable prediction model for Individual Prognosis Or Diagnosis plus Artificial Intelligence (TRIPOD+AI) statement, and potential sources of bias and applicability concerns were considered using the Prediction model Risk Of Bias ASsessment Tool plus Artificial Intelligence (PROBAST+AI) (27, 28).The overall study design and analysis workflow are summarized in **Figure 1**.

**Figure 1:**
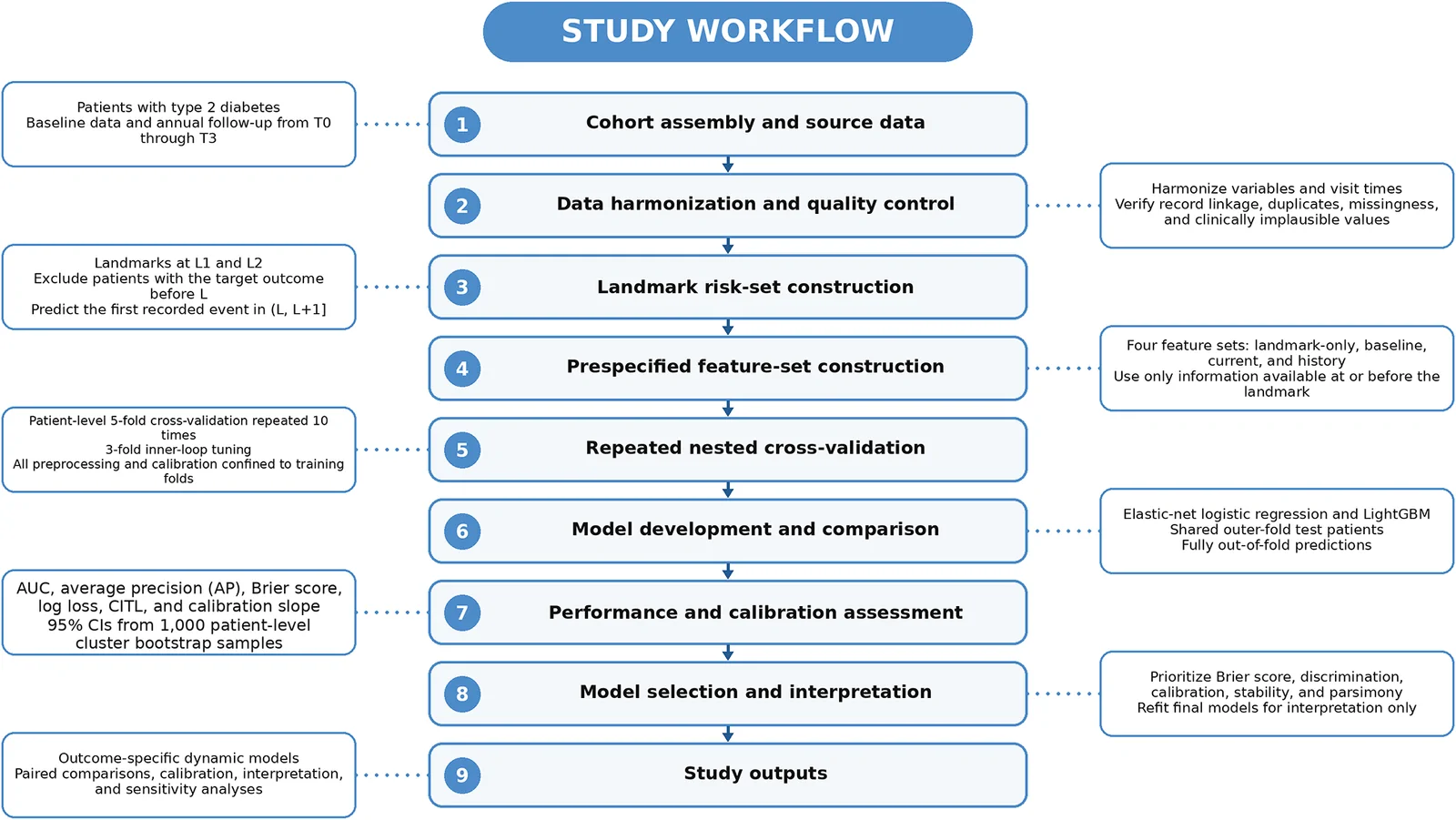
Study design and analysis workflow. Annual visits T0-T3 were used to construct one-year prediction tasks at landmark times L1 (T1) and L2 (T2). For each outcome, patients with a previous recorded diagnosis were excluded from that outcome-specific risk set. Four feature sets, landmark-only, baseline, current, and history, were compared with elastic-net logistic regression and LightGBM using patient-level repeated nested cross-validation. Primary paired comparisons evaluated history versus current information and LightGBM versus elastic-net. Locked models were then refitted for interpretation, and prespecified sensitivity analysis were conducted separately.

## 2 Materials and methods

### 2.1 Study design, participants, and ethics

We conducted a single-center retrospective longitudinal cohort study using electronic medical records and health examination data from the health examination center of a tertiary hospital. In collaboration with the health examination center, we screened patient records with at least three years of available health examination history. Two investigators subsequently conducted a manual review to identify patients for whom type 2 diabetes was the principal documented condition and to assemble the corresponding diabetes-related records together with health examination data from the preceding three years. All direct personal identifiers were removed before analysis, and static and longitudinal records were linked using anonymized patient identifiers. The study was conducted in accordance with the Declaration of Helsinki and was approved by the Ethics Committee of Shenzhen Hospital, Guangzhou University of Chinese Medicine (approval no. GZYLL(KY)-2025-146).

Participants were eligible if they were aged 40 years or older, met the diagnostic criteria for type 2 diabetes specified in the Chinese guideline for the prevention and treatment of diabetes mellitus (2024 edition) (6), had static and longitudinal records that could be matched reliably, had annual records from T0 to T3, and had an ascertainable target-outcome status in the relevant prediction window. The two-investigator manual review was used for cohort eligibility, consistency checking, and data confirmation. It was not an independent clinical outcome-adjudication process. We excluded records that remained non-unique after checking, could not be matched between static and longitudinal data, could not be assigned to the prespecified T0-T3 visits, were insufficient to form adjacent annual prediction windows, or contained unresolved contradictions that prevented outcome ascertainment. Missing predictor values alone were not a patient-level exclusion criterion.

The final cohort comprised 646 patients with complete T0-T3 follow-up, defining the analytic target population as patients with type 2 diabetes who met these criteria and had complete annual T0-T3 records in this study setting. Baseline characteristics of the analytic cohort are summarized in **Table 1**. Cohort size was determined by the eligible data available for analysis rather than by a prespecified model-development sample-size calculation. Because pooled person-landmark records include repeated observations from the same individuals, sample-size interpretation considered the number of unique patients and outcome events together with model complexity rather than the total number of person-landmark records alone. Event counts are reported by outcome and landmark in **Table 2**. Model complexity was controlled through prespecified feature sets, limited landmark interactions, regularization or tuned tree constraints, and patient-level nested cross-validation. Additional information on at-risk patients, events, candidate features, model-selection stability, and model-development stability is provided in **Supplementary Table 6C-9**. Sample-size interpretation followed contemporary prediction-model guidance (29). Complete T0-T3 follow-up was an intentional cohort-design restriction used to provide a common longitudinal source cohort across L1 and L2. It was not required by landmarking itself. T3 was used to ascertain L2-to-T3 outcomes but was not used as a predictor. For L1-to-T2, T3 was not required for predictor construction or outcome ascertainment, so its availability affected cohort eligibility only and made L1 inclusion conditional on subsequent T3 follow-up.

**Table 1:** Baseline characteristics of the study population (n = 646).

Characteristic (unit)	Overall	Missing, n (%)
Demographics and diabetes duration		
Male, n (%)	486 (75.2%)	0 (0.0%)
Age, years	54.5 ± 10.1	0 (0.0%)
Diabetes duration, years	8.9 ± 5.9	0 (0.0%)
Anthropometric measures		
Height, cm	167.3 ± 7.1	0 (0.0%)
Weight, kg	71.1 ± 12.5	41 (6.3%)
Body mass index, kg/m^2^	25.3 ± 3.8	41 (6.3%)
Waist circumference, cm	89.2 ± 9.8	40 (6.2%)
Waist-to-hip ratio, unitless	0.92 ± 0.07	52 (8.0%)
Glycemic and blood pressure measures		
Fasting plasma glucose, mmol/L	7.37 (6.34, 9.31)	42 (6.5%)
Glycated hemoglobin, %	7.8 ± 1.7	54 (8.4%)
Systolic blood pressure, mm Hg	146.6 ± 20.5	48 (7.4%)
Diastolic blood pressure, mm Hg	91.8 ± 12.8	46 (7.1%)
Kidney function and albuminuria		
Urinary albumin-to-creatinine ratio, mg/mmol	0.72 (0.38, 1.44)	71 (11.0%)
Serum creatinine, μmol/L	74.3 ± 14.3	32 (5.0%)
Uric acid, μmol/L	323.7 ± 57.3	39 (6.0%)
Lipid profile		
Total cholesterol, mmol/L	4.85 ± 0.87	41 (6.3%)
Low-density lipoprotein cholesterol, mmol/L	2.96 ± 0.61	40 (6.2%)
High-density lipoprotein cholesterol, mmol/L	1.28 ± 0.25	35 (5.4%)
Triglycerides, mmol/L	1.88 (1.33, 2.49)	49 (7.6%)
Liver enzymes, inflammation, and electrocardiography		
Alanine aminotransferase, U/L	34.5 (24.4, 46.3)	41 (6.3%)
Aspartate aminotransferase, U/L	29.2 (23.5, 35.8)	55 (8.5%)
Gamma-glutamyl transferase, U/L	35.8 (27.2, 43.5)	50 (7.7%)
White blood cell count, ×10^9^/L	6.9 ± 1.3	40 (6.2%)
Abnormal resting electrocardiogram, n (%)	18 (2.8%)	0 (0.0%)

Continuous variables are presented as mean ± standard deviation or median (25th percentile, 75th percentile), and categorical variables as n (%). **Table 1** presents representative baseline characteristics at T0. The complete candidate predictor set and units are provided in **Supplementary Table 1**.

**Table 2:** Event distribution for the five diabetes-related clinical outcomes across landmark prediction windows (combined L1-L2 counts are person-landmark records).

Outcome	L1 records/events	L1 event rate	L2 records/events	L2 event rate	Combined L1-L2 records/events (event rate)
HTN	502/82	16.3%	420/43	10.2%	922/125 (13.6%)
CHD	634/123	19.4%	511/78	15.3%	1145/201 (17.6%)
DKD	637/71	11.1%	566/42	7.4%	1203/113 (9.4%)
DR	641/34	5.3%	607/66	10.9%	1248/100 (8.0%)
MASLD	376/65	17.3%	311/15	4.8%	687/80 (11.6%)

### 2.2 Outcome definitions and landmark risk sets

The outcomes were HTN, CHD, DKD, DR, and MASLD. Each outcome was ascertained from the corresponding clinician-documented diagnosis-status field in the source EHR during routine clinical care, with reference to the relevant cardiovascular, kidney, retinal, and liver guidelines or nomenclature statements (1–9). In the final 646-patient analysis extract, 0 indicated that the target outcome was not recorded as present at that annual assessment, whereas 1 indicated that it was recorded as present. Following the two-investigator eligibility, consistency, and coding review, the final outcome fields contained no missing values and no separate uncertain category. These operational binary fields represent recorded annual outcome status in the source EHR rather than independently adjudicated disease onset. For patients without a prior positive record of the target outcome at landmark L, an event was defined as the first positive outcome record during the interval (L, L + 1]. Accordingly, the prediction target was the next-year risk of a first positive outcome record at an annual assessment rather than the exact timing of disease onset between annual visits.

In practical terms, T0 was the baseline visit, followed by three annual visits T1-T3. T1 and T2 were defined as landmark times L1 and L2. Separate landmark risk sets were then created for each outcome. At landmark L, patients were included if outcome status was ascertainable at L and L + 1 and the target outcome had not been recorded by L. The target was the first recorded outcome during (L, L + 1]. A patient with a previous record of one target outcome left only that outcome-specific risk set and could remain eligible for analysis of other outcomes. In the main analysis, once an outcome was first recorded as positive, subsequent records were treated as positive, with alternative handling examined in sensitivity analysis.

### 2.3 Predictors and data processing

Candidate predictors covered demographics, diabetes duration, anthropometry, glycemia, blood pressure, kidney function and urinary protein, lipids, liver function, inflammation, myocardial injury, and other routine clinical measures. The longitudinal data contained 31 repeated measures. The complete predictor list, units, feature-set membership, longitudinal feature definitions, and outcome-specific exclusions are provided in **Supplementary Table 1**.

We defined four progressively expanded feature sets: landmark-only, baseline, current, and history. The landmark-only set contained landmark-time information. The baseline set additionally included the baseline-fixed predictors sex, baseline age, diabetes duration, and height. The current set then added clinical measurements available at the current landmark visit, together with the current status and count of non-target comorbidities. The history set additionally included prior means and recent changes calculated using information available up to the landmark. For each history variable, the prior mean summarized measurements obtained before the landmark, whereas the recent change was calculated as the landmark value minus the value at the preceding visit. Prespecified interactions with landmark time were included for a small core set of variables in the baseline and richer feature sets. Full definitions are provided in **Supplementary Table 1**. The amount of available longitudinal history naturally differed between L1 and L2 because an additional intermediate visit was available by L2. Accordingly, the history-current comparison reflects the added predictive value of the longitudinal information available at each landmark rather than an identical duration or amount of prior information at both time points.

We harmonized field names and visit timing, checked patient and visit uniqueness, verified linkage between static and longitudinal records, and screened values against prespecified clinically plausible ranges. Variables that could be derived deterministically within the same visit were then calculated, and prespecified right-skewed variables were transformed using log(1+x). Cross-year interpolation and future-visit information were not used to impute earlier missing values. Candidate columns that were structurally all missing or constant in the assembled analysis dataset were removed once before resampling without reference to outcome values. For elastic-net regression, median imputation, missingness indicators, and RobustScaler transformation were fitted within training folds. LightGBM used its native handling of missing values. Candidate predictors were selected on the basis of clinical availability and a prespecified feature dictionary rather than univariable P values or stepwise selection.

To reduce the influence of diagnostic proximity, the main analysis excluded measurements closely related to the contemporaneous diagnosis of the corresponding outcome. These variables were reintroduced in sensitivity analysis. They were already available at the landmark and therefore did not represent future information, but they could inflate apparent performance when a formal diagnosis was recorded later than the underlying clinical state.

### 2.4 Model development and internal validation

After deterministic structural exclusions and removal of outcome-proximal predictors, the outcome-specific feature maps contained 1 landmark-only feature, 7 baseline features, 40–42 current-visit features, and 73–78 history features before fold-specific preprocessing and regularization. Because the L2 MASLD analysis included only 15 events, its landmark-specific estimates were treated as descriptive and were not used for model ranking.

We compared elastic-net logistic regression (30) with LightGBM (31) using patient-level repeated nested cross-validation (32). The outer loop used five folds repeated 10 times to generate out-of-fold (OOF) predictions, and the inner loop used three folds for hyperparameter optimization. Within each repeat, all landmark records from the same patient stayed in the same outer fold. Feature-set and algorithm comparisons therefore used identical outer test patients. Each outer-training set was tuned independently using 30 Optuna trials, with hyperparameters selected by minimizing the mean Brier score across patient-grouped inner-validation folds. Additional validation, seed, and reproducibility settings are summarized in **Supplementary Table 5D**.

Within this setup, probability calibration used sigmoid calibration fitted from OOF predictions generated within the outer training data. Patients in the outer test folds did not contribute to imputation, scaling, tuning, model fitting, or calibration. The primary history-current estimand was calculated separately within elastic-net and LightGBM. Within each paired contrast, the outcome-specific patient risk set, outer test folds, validation splits, preprocessing, tuning framework, calibration procedure, and evaluation records were identical. Each outer fold was independently tuned and calibrated, while models retuned on the complete development data were reserved for interpretation and stability analysis and were not used to generate OOF performance estimates (**Supplementary Tables 6A, B**).

We selected five representative models after candidate OOF evaluation using a prespecified hierarchy that prioritized the Brier score and also considered AUC, AP, calibration, between-repeat stability, and model complexity. Because the same internal-validation results informed both model selection and descriptive performance reporting, performance estimates for the selected representative models were treated as descriptive rather than as unbiased estimates of post-selection performance. The main scientific conclusions therefore focused on the paired history-current and LightGBM-elastic-net contrasts.

To assess uncertainty arising from model selection, we additionally performed a fully nested sensitivity analysis in which all candidate feature-set and algorithm configurations were retuned and selected using only the outer-training data according to the lowest mean inner-fold Brier score, and the selected configuration was then evaluated in the untouched outer-test fold. This analysis assessed the performance and variability of the model-selection procedure and did not alter the descriptive interpretation of the representative-model absolute performance.

To assess model-development stability, we performed 500 patient-level nonparametric bootstrap replicates for each prespecified configuration. Patients were sampled with replacement, retaining duplicate-patient multiplicity. Each replicate repeated structural screening, fold-specific preprocessing, 30-trial inner tuning, model fitting, and sigmoid calibration. Inner tuning and calibration folds were grouped by source patient identifier to prevent duplicated source patients from crossing folds. Performance was evaluated only in out-of-bag patients. Original-cohort predictions were used only for instability summaries. We report medians, 2.5th-97.5th percentile ranges, and favorable-direction proportions for paired contrasts (33).

To confirm temporal isolation of L1 construction from T3 values, we performed an L1 T3-masking audit in which all T3 dynamic values were deliberately altered before reconstructing the L1 datasets. Patient membership, event labels, predictor columns, and predictor values were compared with the original datasets. This audit assessed temporal data leakage but not selection related to the complete T0-T3 cohort restriction.

### 2.5 Model evaluation, statistical analysis, and interpretation

Models were evaluated at L1, L2, and across pooled L1-L2 records. The main performance measures were the area under the receiver operating characteristic curve (AUC), average precision (AP), Brier score, calibration-in-the-large, and calibration slope (34). Log loss and the Brier skill score were supplementary measures.

For each metric, values were first calculated within each outer cross-validation repeat and then summarized across the 10 repeats. Ninety-five percent confidence intervals (CIs) were estimated using 1,000 patient-level cluster bootstrap samples applied to the completed repeated nested-CV OOF predictions. During resampling, patients were sampled with replacement while all of their associated landmark records were retained together. Model fitting, hyperparameter tuning, and calibration were not repeated within these 1,000 resamples. Accordingly, the intervals quantify patient-level resampling variability around the realized repeated-CV OOF predictions rather than uncertainty from repeating the entire model-development procedure. Additional uncertainty related to model development and selection was evaluated separately using fully nested model-selection and model-development bootstrap analysis.

The incremental value of longitudinal information was defined as the paired history-minus-current difference within each algorithm, with elastic-net and LightGBM evaluated separately. Algorithm differences were defined as paired LightGBM-minus-elastic-net differences within each feature set. Interpretation considered paired differences and their 95% confidence intervals together with AUC, AP, Brier score, calibration, and sensitivity analysis. Repeated-CV paired comparisons and full model-development bootstrap summaries were reported separately because they address different sources of uncertainty: the former summarize performance differences based on the realized repeated-CV OOF predictions, whereas the latter assess variability when model development is repeated across bootstrap samples. No formal multiplicity adjustment was applied across the multiple outcomes, feature sets, algorithms, and metrics. Confidence intervals were therefore interpreted primarily as measures of effect magnitude and precision, while secondary comparisons were considered exploratory.

Once internal validation was complete and the interpretation plan had been locked, we refitted the models used for interpretation in the full development data. Elastic-net models were summarized by regularized coefficients and patient-level bootstrap selection stability. LightGBM models were interpreted using Shapley additive explanations (SHAP), global importance, and ranking stability (35). These analysis describe predictive associations rather than causal effects. For exploratory decision-curve analysis, the 10 repeated OOF probabilities for each patient-landmark record were averaged before net benefit was calculated. Results are shown in **Supplementary Figure 1** (36) and were not used for representative-model selection. Because clinical actions and externally validated decision thresholds were not prespecified, decision-curve findings were interpreted as exploratory. We also examined several sensitivity scenarios, including alternative handling of nonmonotonic outcome records, reintroduction of contemporaneous variables closely related to diagnosis, removal of other comorbidity-status predictors, and extension of the landmark range to pooled landmark times 0-2 (L0-L2). All data processing, model development, internal validation, and statistical analysis were performed in Python 3.9.25 using pandas 1.5.3, NumPy 1.23.5, SciPy 1.10.1, scikit-learn 1.5.2, LightGBM 3.3.2, and Optuna 3.2.0. Search spaces and fixed implementation settings are detailed in **Supplementary Methods 1.2.6–1.2.7**, and reproducibility settings are summarized in **Supplementary Table 5D**. Reporting followed TRIPOD+AI and the Strengthening the Reporting of Observational Studies in Epidemiology statement, with PROBAST+AI used to consider potential bias (27, 28, 37).

## 3 Results

### 3.1 Study population, data quality, and landmark risk sets

A total of 646 patients contributed 2,584 annual follow-up records from T0 to T3. At baseline, 486 participants (75.2%) were male, the mean age was 54.5 ± 10.1 years, and the mean duration of diabetes was 8.9 ± 5.9 years. Other baseline characteristics are shown in **Table 1**.

After these data checks, static and longitudinal records were matched successfully. Across the 31 longitudinal variables and four visits, the median missingness after data cleaning was 6.2%, with a maximum of 12.4%. Missing-data handling was restricted to training folds. Depending on the outcome, L1 comprised 376–641 person-landmark records and L2 comprised 311–607 records. Counts for pooled L1-L2 represent person-landmark records rather than unique patients. Risk-set composition and event distribution are shown in **Table 2**, and data-quality and OOF-integrity audits are summarized in **Supplementary Table 5**.

### 3.2 Internal validation performance of representative models

Across the five representative models selected after candidate OOF evaluation, pooled L1-L2 OOF AUCs ranged from 0.722 to 0.952 and were interpreted descriptively. Calibration-in-the-large was close to 0, and calibration slopes ranged from 0.936 to 1.008. AUC was lower at L2 than at L1 for HTN and DKD. The L2 MASLD analysis included only 15 events, so its landmark-specific AP and calibration estimates were also interpreted descriptively. **Table 3** and **Figure 2** summarize the internal-validation performance, and **Supplementary Figure 1** presents the exploratory decision curves.

**Table 3:** Descriptive internal-validation performance of the representative models for the five outcomes.

Outcome	Representative model	AUC (95% CI)	AP (95% CI)	Brier score (95% CI)	Calibration-in-the-large (95% CI)	Calibration slope (95% CI)
HTN	history LightGBM	0.785 (0.741, 0.825)	0.389 (0.321, 0.469)	0.100 (0.085, 0.115)	-0.017 (-0.227, 0.173)	1.006 (0.829, 1.209)
CHD	current LightGBM	0.722 (0.688, 0.755)	0.335 (0.289, 0.396)	0.132 (0.119, 0.146)	-0.011 (-0.158, 0.155)	1.008 (0.848, 1.195)
DKD	baseline Elastic-net	0.762 (0.711, 0.809)	0.297 (0.227, 0.380)	0.076 (0.065, 0.087)	-0.003 (-0.229, 0.174)	0.994 (0.792, 1.222)
DR	current Elastic-net	0.921 (0.899, 0.941)	0.566 (0.481, 0.652)	0.050 (0.041, 0.059)	-0.016 (-0.286, 0.239)	0.936 (0.815, 1.103)
MASLD	history LightGBM	0.952 (0.933, 0.969)	0.740 (0.649, 0.825)	0.053 (0.041, 0.067)	0.010 (-0.351, 0.370)	0.960 (0.830, 1.142)

Point estimates are the mean metric values across 10 outer cross-validation repetitions. The 95% confidence intervals were estimated using 1,000 patient-level cluster bootstrap samples. AUC, AP, Brier score, calibration-in-the-large, and calibration slope are dimensionless metrics. Performance of the representative models is reported for descriptive internal validation.

**Figure 2:**
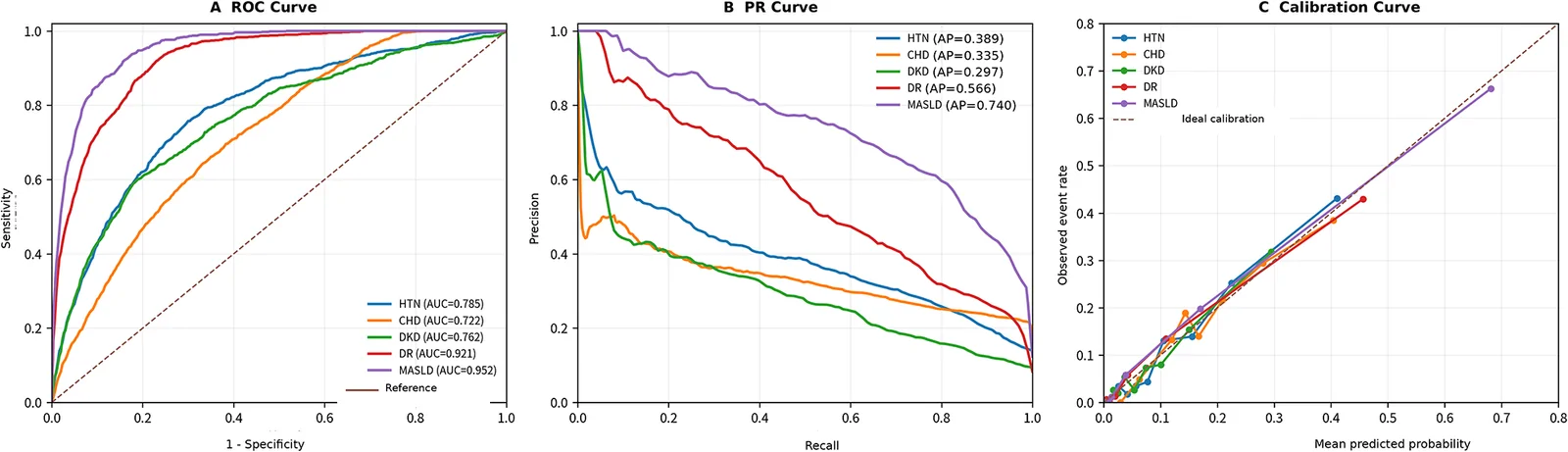
Discrimination and calibration of the five representative models. Panel **(A)** shows the mean receiver operating characteristic curves and panel **(B)** the mean precision-recall curves across 10 repeated out-of-fold predictions. Panel **(C)** shows calibration by predicted-probability quantiles. Curves are displayed for the representative models for HTN, CHD, DKD, DR, and MASLD.

### 3.3 Incremental value of longitudinal history and algorithms

The paired within-algorithm comparisons showed the clearest positive differences for HTN and MASLD. For HTN, history-current ΔAUC was 0.097 (95% confidence interval 0.057 to 0.136) with elastic-net and 0.118 (0.081 to 0.158) with LightGBM. For MASLD, the corresponding pooled L1-L2 differences were 0.022 (0.002 to 0.042) and 0.025 (0.009 to 0.041). CHD, DKD, and DR showed no comparable pooled improvement with either algorithm. **Table 4** and **Figure 3** present all 10 within-algorithm contrasts.

**Table 4:** Within-algorithm paired performance differences for history versus current feature sets.

Outcome	Algorithm	ΔAUC (95% CI)	ΔAP (95% CI)	ΔBrier (95% CI)	Interpretation
HTN	Elastic-net	0.097 (0.057, 0.136)	0.069 (0.027, 0.118)	-0.006 (-0.012, -0.002)	Positive pooled difference
HTN	LightGBM	0.118 (0.081, 0.158)	0.140 (0.090, 0.192)	-0.012 (-0.018, -0.007)	Positive pooled difference
CHD	Elastic-net	0.001 (-0.011, 0.013)	-0.004 (-0.027, 0.012)	0.000 (-0.001, 0.002)	No clear pooled difference
CHD	LightGBM	-0.003 (-0.012, 0.008)	-0.002 (-0.018, 0.014)	0.000 (-0.001, 0.001)	No clear pooled difference
DKD	Elastic-net	-0.010 (-0.021, 0.001)	-0.011 (-0.031, 0.008)	0.001 (0.000, 0.002)	No clear pooled difference
DKD	LightGBM	-0.001 (-0.015, 0.013)	0.000 (-0.021, 0.019)	0.000 (-0.001, 0.001)	No clear pooled difference
DR	Elastic-net	-0.001 (-0.007, 0.005)	-0.018 (-0.044, 0.010)	0.001 (-0.001, 0.003)	No clear pooled difference
DR	LightGBM	-0.001 (-0.007, 0.007)	0.005 (-0.019, 0.024)	0.000 (-0.002, 0.001)	No clear pooled difference
MASLD	Elastic-net	0.022 (0.002, 0.042)	0.043 (-0.003, 0.094)	-0.005 (-0.010, 0.000)	Positive pooled difference
MASLD	LightGBM	0.025 (0.009, 0.041)	0.079 (0.030, 0.121)	-0.008 (-0.013, -0.002)	Positive pooled difference

Each row is a within-algorithm paired comparison of history minus current. The outcome-specific risk set, outer validation split, preprocessing, tuning framework, calibration procedure, and evaluation records were held constant within each contrast. Point estimates are means of repeat-specific paired differences across the 10 outer cross-validation repetitions. The 95% confidence intervals were obtained by patient-level cluster bootstrap of the completed repeated-CV OOF predictions and were not adjusted for multiplicity. Positive ΔAUC and ΔAP values and negative ΔBrier values favor history.

**Figure 3:**
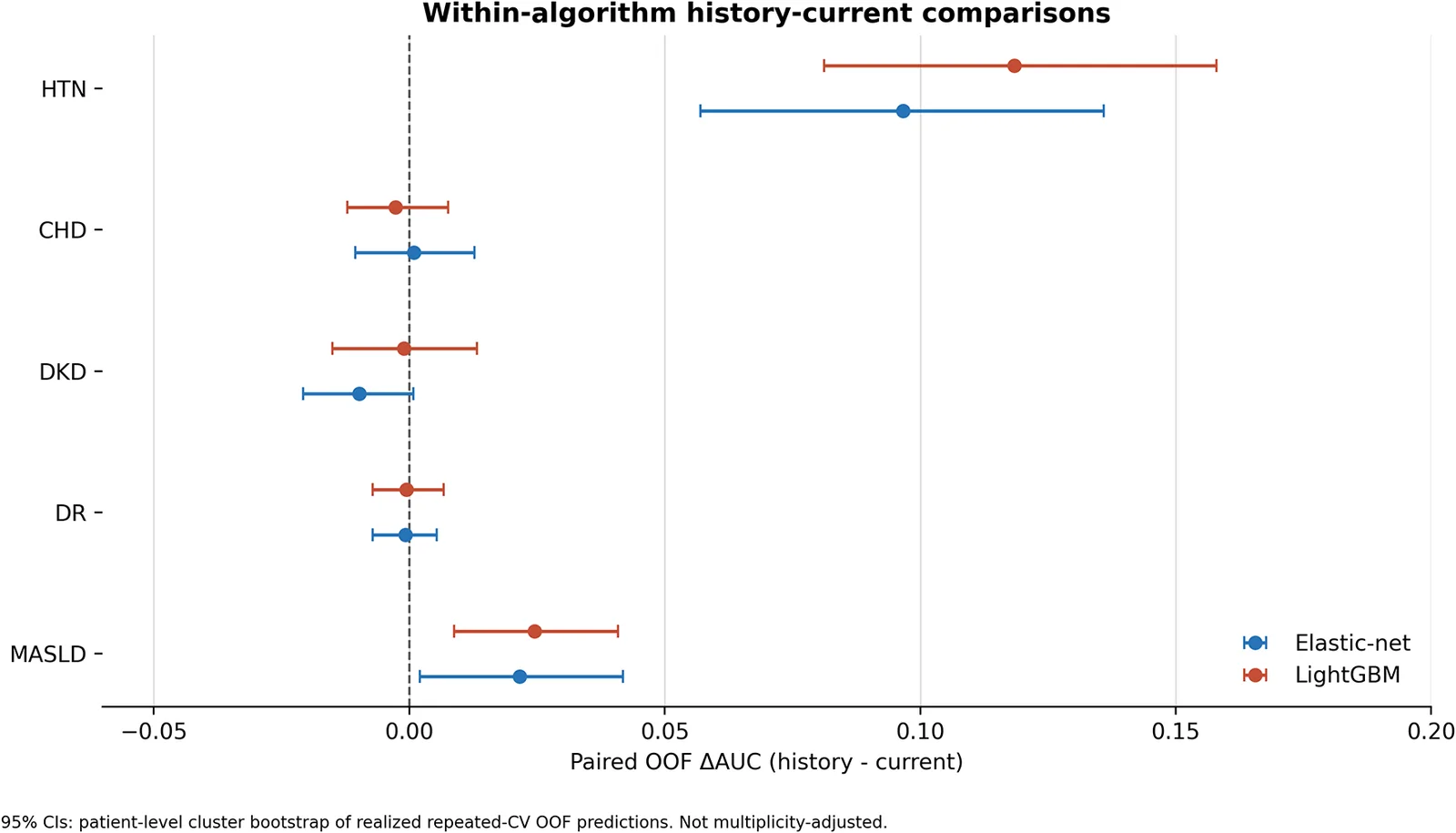
Paired OOF delta AUC for history versus current feature sets within each algorithm. Points show the mean paired delta AUC calculated as history minus current, and horizontal lines show 95% confidence intervals. Elastic-net and LightGBM are shown separately for each outcome. Within each contrast, the outcome-specific risk set, outer validation split, preprocessing, tuning framework, calibration procedure, and evaluation records were identical. Confidence intervals were obtained using patient-level cluster bootstrap resampling of the realized repeated-CV OOF predictions and were not multiplicity-adjusted. The vertical reference line at 0 indicates no difference. Positive values favor history.

By contrast, algorithm differences were smaller and outcome dependent. LightGBM had a modest AUC advantage for CHD and MASLD, whereas elastic-net achieved higher AP for DR. Performance was similar between the two algorithms for HTN and DKD. **Supplementary Table 2** provides the complete primary-analysis performance estimates and paired algorithm comparisons.

In the fully nested model-selection analysis, the representative configuration was most frequently selected for four of the five outcomes, including HTN history-LightGBM in 47 of 50 outer fits and MASLD history-LightGBM in 49 of 50. CHD showed greater selection variability, with baseline-LightGBM selected in 28 of 50 fits and current-LightGBM in 15 of 50. Pooled AUCs from the fully nested procedure ranged from 0.715 to 0.950 (**Supplementary Table 7**).

Model-development bootstrap analysis provided complementary information on development-sample stability and was interpreted separately from the repeated-CV paired comparisons. Across 500 patient-level bootstrap replicates, median pooled out-of-bag AUCs for the representative models ranged from 0.713 to 0.952. For the representative LightGBM comparisons, the history-current AUC difference was positive in all 500 replicates for HTN, with a median difference of 0.117, and in 480 of 500 replicates for MASLD, with a median difference of 0.025. Detailed stability results are provided in **Supplementary Tables 8, 9** and **Supplementary Figure 2–4**.

### 3.4 Model interpretation and stability

Eight locked models underwent stability analysis: five outcome-specific representative models and three elastic-net comparison models. For HTN, prior triglycerides, prior uric acid, and recent change in serum creatinine ranked highest. The CHD model emphasized diabetes duration, age, and current serum creatinine. For MASLD, prior and current body mass index and recent change in total cholesterol were most prominent. The elastic-net models for DKD and DR consistently retained age or diabetes duration, and the DR model also retained current urinary albumin-to-creatinine ratio. These are predictive associations, not causal effects. **Supplementary Table 3** gives the detailed coefficients, SHAP rankings, and bootstrap stability results.

### 3.5 Sensitivity analysis

We evaluated the outcome-specific representative configurations under several sensitivity scenarios. Across four alternative L1–L2 analysis, the history-current AUC difference remained positive for HTN and MASLD, whereas CHD, DKD, and DR showed no comparable positive pattern. These sensitivity analysis were configuration-specific and did not replace the primary within-algorithm contrasts reported in **Table 4**. When the landmark range was extended to pooled L0–L2, the history-current differences for HTN and MASLD remained positive but were attenuated. Because L0 had no preceding longitudinal history, this analysis was considered exploratory (**Table 5**). Detailed sensitivity results are reported in **Supplementary Table 4**.

**Table 5:** Sensitivity analysis for the outcome-specific representative-algorithm history-current comparisons.

Outcome	Main-analysis algorithm	Main-analysis ΔAUC (95% CI)	Range of ΔAUC point estimates across 4 alternative L1-L2 scenarios	Pooled L0-L2 ΔAUC (95% CI)	Interpretation
HTN	LightGBM	0.118 (0.081, 0.158)	0.075 to 0.115	0.031 (0.014, 0.047)	Attenuated gain
CHD	LightGBM	-0.003 (-0.012, 0.008)	-0.007 to -0.002	-0.013 (-0.023, -0.005)	No clear incremental gain
DKD	Elastic-net	-0.010 (-0.021, 0.001)	-0.006 to 0.001	-0.008 (-0.023, 0.008)	No clear incremental gain
DR	Elastic-net	-0.001 (-0.007, 0.005)	-0.002 to -0.001	-0.014 (-0.025, -0.003)	No clear incremental gain
MASLD	LightGBM	0.025 (0.009, 0.041)	0.025 to 0.029	0.005 (0.001, 0.008)	Attenuated gain

ΔAUC was calculated as history minus current. Alternative scenarios included alternative handling of nonmonotonic outcome records, inclusion of contemporaneous variables closely related to diagnosis, and alternative comorbidity-predictor configurations. Pooled L0-L2 represents an exploratory analysis with an expanded landmark range.

A separate sensitivity analysis looked at contemporaneous variables that were closely related to diagnosis. When these variables were reintroduced, AUC increased from 0.722 to 0.974 for the current-LightGBM CHD model and from 0.767 to 0.972 for the current-elastic-net DKD model. This suggests that diagnostic-proximal variables can materially increase apparent performance. Across the algorithm sensitivity analysis, the LightGBM advantage for CHD varied by scenario, the LightGBM advantage for MASLD was more consistent, and the AP advantage of elastic-net for DR was maintained in most scenarios. Detailed results are provided in **Supplementary Table 4**.

## 4 Discussion

### 4.1 Principal findings

Overall, the incremental value of longitudinal history was outcome specific. In paired comparisons that held the algorithm and validation workflow constant, longitudinal history showed a clear pooled internal-validation gain for HTN with both algorithms and a smaller but positive pooled gain for MASLD. CHD, DKD, and DR showed limited additional predictive value from the historical summaries evaluated here. The incremental value of model complexity was likewise outcome dependent. These findings support outcome-specific use of longitudinal information within this single-center complete-follow-up cohort, while external validation is needed to assess transportability.

This distinction matters because many previous prediction studies have focused on a single outcome or a fixed prediction origin (10–21). In contrast, our analysis compared information depth and algorithm within the same patients, one-year prediction windows, and validation splits rather than simply identifying the model with the highest AUC. This paired internal-validation framework provides a consistent basis for assessing whether additional longitudinal information or model complexity adds predictive value within the present dataset. Independent external validation is still required to determine whether these outcome-specific patterns generalize across populations, institutions, and time periods.

### 4.2 Outcome-specific value of longitudinal history

For HTN, the paired pooled OOF history-current AUC differences were 0.097 with elastic-net and 0.118 with LightGBM, both in favor of the history representation. The differences remained positive across the alternative L1–L2 sensitivity analysis. Prior triglycerides, prior uric acid, and recent change in serum creatinine ranked prominently in the SHAP analysis. These longer-term metabolic and renal measures may help distinguish patients with similar current profiles but different preceding trajectories. Baseline blood pressure was relatively high in this cohort, and the outcome was defined by the first recorded occurrence at an annual assessment. In a sensitivity analysis, reintroducing landmark systolic and diastolic blood pressure markedly increased AUC, highlighting the importance of predictor timing relative to outcome documentation. The observed history gain should therefore be interpreted as improved prediction of recorded HTN occurrence within the present study framework rather than as evidence about the exact timing of disease onset. Previous studies in type 2 diabetes have also identified metabolic and renal measures as useful predictors of HTN (17, 38).

For MASLD, the pooled L1–L2 repeated-CV OOF history-current AUC differences were 0.022 with elastic-net and 0.025 with LightGBM. For the representative LightGBM comparison, the history-current difference was positive in 480 of 500 model-development bootstrap replicates. Together, these findings support a modest pooled internal-validation signal, although they do not demonstrate consistent benefit at each landmark. In particular, the L2 analysis included only 15 events, so its landmark-specific estimates of discrimination and calibration should be interpreted descriptively. Prior mean body mass index, current body mass index, and recent change in total cholesterol remained prominent in the model-interpretation analysis, consistent with the contribution of adiposity and metabolic burden to MASLD (4, 5, 7, 21). Longitudinal adiposity and metabolic information may therefore provide predictive context beyond measurements from a single visit.

In contrast, CHD, DKD, and DR did not show a comparable history gain. Over a one-year prediction window, recent measurements and stable baseline characteristics may already capture much of the useful short-term information. The present findings do not imply that longer clinical histories are uninformative; rather, the prior means and recent changes evaluated here provided limited additional OOF predictive value for these three outcomes. Denser or more frequent longitudinal measurements may allow future studies to evaluate richer trajectory representations.

### 4.3 Algorithm comparison and model interpretation

The same caution applies to algorithm choice: more complex modeling was not consistently better. With the current feature set for CHD, the OOF AUC was 0.024 higher with LightGBM than with elastic-net, but the size of this advantage changed across sensitivity analysis. For MASLD with the history set, the LightGBM advantage was more consistent. The algorithms performed similarly for HTN, while elastic-net was comparable or higher on selected performance metrics for DKD and DR. When predictive differences are small, model simplicity, interpretability, and downstream validation burden should also be considered.

Model interpretation showed a similar outcome-specific pattern. The HTN and MASLD models assigned greater predictive importance to longer-term metabolic measures and recent changes, whereas the CHD model was influenced primarily by diabetes duration, age, and current serum creatinine. Age and diabetes duration were retained consistently in the DKD model, and the DR model also retained current urinary albumin-to-creatinine ratio. These patterns are broadly consistent with established clinical knowledge (2, 3, 8, 9, 14–16, 20, 39, 40). However, SHAP importance and regularized coefficients represent model-specific predictive contributions and should not be interpreted as independent or causal effects.

### 4.4 Clinical relevance, novelty, and limitations

From a practical perspective, the study compared four information depths and two algorithms in the same patients using a common prediction horizon and internal-validation framework, allowing their incremental value to be assessed consistently. The paired results support retaining longitudinal information for HTN and suggest a more modest pooled benefit for MASLD, whereas simpler information configurations may be reasonable starting points for CHD, DKD, and DR. The fully nested model-selection analysis and 500-replicate model-development bootstrap provided complementary evidence on internal stability. Overall, these findings indicate that the value of additional longitudinal information and model complexity depends on the specific prediction task.

Previous work has evaluated prediction tools embedded in electronic medical records or tested them in independent populations (41, 42), but neither step has yet been completed for the present models. Potential use for annual risk updating or prioritization for closer assessment would therefore require independent validation, recalibration where appropriate, prespecified clinical actions and thresholds, and prospective evaluation of clinical impact. The present models are intended to complement rather than replace established disease-specific screening and diagnostic procedures. Accordingly, the decision-curve analysis was interpreted as exploratory.

Several considerations should be taken into account when interpreting these findings. First, this single-center study was restricted to patients with complete T0–T3 follow-up, defining the analytic target population and potentially affecting representativeness through longitudinal data completeness (43). Eligibility for L1 was therefore conditional on subsequent T3 follow-up availability, although the T3-masking audit confirmed that T3 values did not enter L1 predictor or outcome construction. Because records of otherwise eligible patients without T3 follow-up and the full upstream screening denominator were not retained, a window-specific L1 cohort could not be reconstructed and the magnitude of selection related to follow-up completeness could not be quantified. Second, some outcome-landmark strata were sparse, particularly MASLD at L2 with 15 events, limiting landmark-specific interpretation. Third, annual EHR outcome-status fields did not identify exact disease-onset timing between visits, and diagnostic proximity may partly explain the large performance increases observed when diagnosis-proximal variables were reintroduced. Finally, because representative models were selected after candidate OOF evaluation, their absolute OOF performance was interpreted descriptively. The fully nested model-selection and model-development bootstrap analysis provided complementary evidence on stability, but independent external validation, including temporal and multicenter validation, remains necessary before clinical application.

### 4.5 Conclusion

Overall, this pooled landmarking study found outcome-specific differences in the internally validated incremental value of longitudinal information and model complexity. Within-algorithm paired comparisons showed the clearest pooled gain from longitudinal history for HTN and a more modest pooled gain for MASLD; the MASLD finding should be interpreted primarily at the pooled L1–L2 level because the L2 analysis included few events. CHD, DKD, and DR showed limited additional predictive value from the historical summaries evaluated here, while algorithm differences were modest and outcome dependent. These findings support outcome-specific evaluation of information depth and modeling strategy within this single-center complete-follow-up cohort. Independent external validation, recalibration where appropriate, and prospective assessment of clinical utility are required before clinical application.

## Data Availability

The raw data supporting the conclusions of this article will be made available by the authors, without undue reservation.
