# Supplementary material for: Outcome-specific value of longitudinal clinical information and model complexity for dynamic prediction of type 2 diabetes-related outcomes: a pooled landmarking study
Source: Front Endocrinol (Lausanne). 2026 Oct 2;17:1964592. doi: 10.3389/fendo.2026.1964592 (PMC13671971; doi:10.3389/fendo.2026.1964592)
Supplement: Supplementary file 1 [file DataSheet1.pdf]

Supplementary Material

1 Supplementary Data

1.1 Supplementary Methods

Feature construction. The primary pooled landmark analysis used L1 and L2 as landmark times. The landmark-only set represented landmark time. The baseline set added four baseline-fixed predictors, comprising sex, baseline age, diabetes duration, and height. The current set further incorporated prespecified clinical measurements available at the landmark together with the current status and count of non-target comorbidities. The history set additionally included two longitudinal summaries for prespecified variables. Recent change (recent_delta) was defined as the transformed value at L minus the transformed value at L-1, while prior mean (prior_mean) was calculated from available transformed values from T0 through L-1. Predictor construction used only information available at or before the relevant landmark.

Internal validation and performance estimation. Repeated nested cross-validation was conducted at the patient level using five outer folds repeated 10 times and three inner folds for tuning. Within each repeat, all records from the same patient remained in the same outer fold. Candidate columns that were structurally all missing or constant in the assembled analysis data were removed before resampling based on predictor structure alone. Subsequent data-adaptive preprocessing, hyperparameter tuning, model fitting, and sigmoid calibration were confined to the corresponding training data. Within each outer-training set, tuned hyperparameters were held fixed while raw probabilities for calibration were generated by a separately seeded three-fold patient-level OOF procedure. An outer-fold-specific logistic sigmoid calibrator was then fitted and applied to predictions from the corresponding final outer-training refit. Performance estimates were calculated within each outer-CV repeat and averaged across the 10 repeats. Ninety-five percent confidence intervals were estimated with 1,000 patient-level cluster bootstrap samples applied to the completed repeated-CV OOF prediction records. These intervals summarize patient-level resampling variability around the realized OOF predictions.

Exploratory decision-curve analysis. Decision curves were generated from repeated-CV OOF probabilities after averaging the 10 OOF probabilities available for each patient-landmark record. Representative-model selection was completed independently of this supplementary analysis. Net benefit was evaluated over a computational threshold range of 0.01 to 0.50, while Supplementary Figure 1 displays thresholds from 0.01 to 0.30 to provide greater visual resolution across the range most relevant to the observed one-year event rates.

Sensitivity analyses. In the primary analysis, a first positive target-outcome record was carried forward to subsequent visits. Four alternative L1-L2 scenarios were examined. These retained outcome records as observed, excluded patients with a later positive-to-negative status change, reintroduced the prespecified contemporaneous direct diagnostic proxies, or removed the current status and count of non-target comorbidities. The sensitivity analyses used five outer repeats and 15 tuning trials. A separate exploratory analysis extended the pooled landmark range to L0, L1, and L2.

1.2 Additional methodological details

1.2.1 Operational outcome ascertainment and measurement considerations

All five outcomes were defined using the corresponding annual EHR outcome-status fields recorded during routine clinical care. In the final 646-patient extract, every annual outcome field contained one of two retained values: 0 indicated that the target outcome was not recorded as present at that annual assessment, whereas 1 indicated that it was recorded as present. Following the two-investigator eligibility, consistency, and coding review, the final outcome fields contained no missing values, no other numeric values, and no separate uncertain category across 2,584 annual records. These operational states represent recorded annual outcome status in the source EHR rather than independently adjudicated disease onset, and the annual assessments do not determine the exact timing of onset between visits. For patients without a prior positive record at landmark L, the operational event was the first annual assessment during (L, L+1] at which the outcome status was 1. After the first positive outcome record, the primary analytic coding carried the positive status forward to subsequent visits. A small number of nonmonotonic annual recording patterns were observed: one CHD patient and eight MASLD patients had a later positive-to-negative status change, affecting 2 CHD and 11 MASLD annual status cells, respectively. Because these fields reflected recorded annual status rather than independently adjudicated disease trajectories, such changes were not interpreted as evidence of disease reversal. Sensitivity analyses retained the annual status as recorded and separately excluded patients with a later positive-to-

negative change. The two-investigator review supported cohort eligibility, consistency checking, and coding confirmation and was not an independent clinical adjudication process.

1.2.2 Cohort selection and available cohort information

The analysis cohort comprised 646 patients contributing 2,584 annual records from T0 through T3. Complete T0-T3 follow-up was an intentional eligibility restriction and was not required by the landmarking method itself. The design provided a common longitudinal source cohort for evaluating accumulated clinical information across L1 and L2, while outcome-specific landmark risk sets differed according to prior outcome status. T3 was used to ascertain outcomes for the L2-to-T3 window but was not used as a predictor for either landmark task. For L1-to-T2, T3 availability affected eligibility only. The analytic target population was therefore patients with type 2 diabetes who met the study criteria and had complete annual T0-T3 records in this setting. The research dataset was assembled only for patients meeting this criterion. It contains final-cohort data, landmark risk-set counts, outcome event counts, missingness summaries, and OOF-integrity checks, but not the full upstream screening denominator or records for otherwise eligible patients without T3 follow-up. An expanded window-specific L1 cohort cannot therefore be reconstructed, and selection related to follow-up completeness can be described but not quantified from the available study data.

L1 T3-masking data-flow audit. To test whether future T3 information entered L1 construction within the fixed cohort, every T3 dynamic value was deliberately altered and all L1 datasets were rebuilt for five outcomes and four feature sets. All 20 reconstructed datasets were identical to their original versions in patient membership, event labels, predictor columns, and predictor values. This confirms temporal isolation of L1 construction from T3 values. It does not remove or quantify selection caused by restricting the source cohort to patients with complete T0-T3 follow-up.

1.2.3 Candidate model grid and inferential framework

The candidate model grid crossed four prespecified feature sets with two prespecified algorithms for each outcome and landmark scenario. Complete candidate OOF results are reported in Supplementary Table 2 before the five representative models are summarized. The primary history-current estimand was calculated separately within elastic-net and LightGBM. Each paired contrast held the outcome-specific patient risk set, outer validation split, preprocessing, tuning framework, calibration procedure, and evaluation records constant. The representative-algorithm values highlighted in the earlier summary tables are a subset of these 10 primary contrasts, not competing estimates of the same algorithm-specific estimand. Absolute performance of the representative models and the corresponding SHAP or coefficient-stability summaries are post-selection descriptive results.

Fully nested selection and model-development stability extension. A fully nested sensitivity analysis re-tuned all four feature sets and both algorithms within each outer-training set. Candidate selection used the mean inner-fold Brier score, with exact ties resolved by a prespecified deterministic order, and outer-test metrics were reserved for final evaluation. A separate 500-replicate patient bootstrap used the same landmark datasets and repeated structural screening, fold-specific preprocessing, 30-trial inner tuning, fitting, calibration, and out-of-bag prediction. The stability grid comprised 20 comparison configurations, covering five outcomes by current or history features by elastic-net or LightGBM, plus the DKD baseline-elastic-net representative configuration. This yielded 21 configurations per replicate. Bootstrap development samples retained patient multiplicity, and inner tuning and calibration folds were grouped by source patient identifier. Out-of-bag performance was evaluated in out-of-bag patients, while original-cohort predictions were retained for development-instability summaries. All 500 replicates completed across all 21 configurations, yielding 10,500 complete configuration records. Together, these analyses characterize internal model-selection and model-development stability. External and temporal validation remain separate future evaluation steps.

1.2.4 Candidate feature dimensions and event distribution

The outcome-specific candidate sets contained 1 landmark-only feature, 7 baseline features, 40-42 current features, and 73-78 history features across the five outcomes after deterministic structural exclusions and outcome-specific proxy removal. These counts describe candidate dimensions before fold-specific preprocessing and regularization, while the numbers of unique patients and events provide the most relevant context for model complexity. The pooled analyses included 80 MASLD events and 125-201 events for the other outcomes. The L2 MASLD task contained 15 events with a history dimension of up to 78 candidate features, so its landmark-specific estimate is presented descriptively. Model ranking relies on the pooled L1-L2 analyses.

1.2.5 Computational reproducibility

The reported outputs were checked against the retained analysis code, software environment, tuning specifications, and validation records. Prespecified checks covered six scenarios, eight interpretation models, and 100 interpretation-

stability refits per model. These checks strengthen computational traceability of the reported outputs and complement the statistical validation analyses.

1.2.6 Hyperparameter search spaces for the primary analysis

The primary analysis used 30 Optuna trials within each inner-training procedure. Hyperparameters were selected by minimizing the mean Brier score across patient-grouped inner-validation folds using the Optuna Tree-structured Parzen Estimator sampler. For elastic-net, C was searched from 0.001 to 20.0 on a logarithmic scale and l1_ratio from 0.0 to 1.0. For LightGBM, the search ranges were n_estimators 60-450, learning_rate 0.01-0.12 on a logarithmic scale, num_leaves 5-25, max_depth 2-6, min_child_samples 25-120, subsample 0.65-1.00, colsample_bytree 0.55-1.00, reg_alpha 0.00001-20.0 on a logarithmic scale, reg_lambda 0.00001-50.0 on a logarithmic scale, and min_split_gain 0.0-1.0. The subsample_freq parameter was fixed at 1 whenever subsampling was used.

1.2.7 Fixed implementation settings

Elastic-net used median imputation with missingness indicators and RobustScaler before LogisticRegression with penalty=elasticnet, solver=saga, max_iter=8000, and random_state=20260720. LightGBM used native missing-value handling with objective=binary, boosting_type=gbdt, deterministic=True, force_col_wise=True, and random_state=20260720. Deterministic task-specific seeds derived from 20260720 were used for outer and inner split generation, Optuna sampling, calibration splitting, and bootstrap procedures. Candidate columns that were structurally all missing or constant were removed before resampling based on predictor structure. Data-adaptive imputation and scaling were fitted within the corresponding training folds. Outer validation used five patient-level folds repeated 10 times, and inner tuning used three folds. For calibration, a separate derived seed generated three patient-level inner OOF folds after tuning. Models with the selected hyperparameters were refitted across those folds to obtain one raw OOF probability for every outer-training observation. The sensitivity scenarios used the reduced tuning budget specified in the main Methods.

1.2.8 Statistical rationale and methodological support

The pooled landmark framework was based on the dynamic prediction approach described by van Houwelingen, in which risk prediction is updated at prespecified landmark times among individuals who remain at risk (van Houwelingen, 2007). The internal validation strategy followed the principles of nested cross-validation proposed by Varma and Simon, with hyperparameter tuning performed independently from outer-fold performance estimation (Varma and Simon, 2006). Reporting and methodological assessment were guided by current recommendations for prediction model development and evaluation, including TRIPOD+AI and PROBAST+AI (Collins et al., 2024; Moons et al., 2025). Sample size considerations were informed by Riley et al., emphasizing the joint consideration of event frequency, candidate predictor complexity, anticipated model performance, calibration precision, and shrinkage rather than relying on a fixed events-per-variable rule (Riley et al., 2020). The model-development bootstrap procedure was based on the framework described by Riley and Collins, which evaluates the stability of predictive performance and calibration through repeated model development under resampled datasets (Riley and Collins, 2023). These methodological references informed the analytical framework and the interpretation of model performance in the present study.

1.2.9 Sparse-event interpretation and post-selection reporting

The pooled L1-L2 risk sets contained 125 HTN, 201 CHD, 113 DKD, 100 DR, and 80 MASLD events. At L2, MASLD had 15 events, so all L2 MASLD discrimination and calibration estimates are descriptive and cannot independently establish a stable landmark-specific effect. The principal MASLD history-current interpretation is therefore based on the pooled L1-L2 internal-validation comparison, with L1 and L2 results reported only as descriptive context. Five representative models were selected after complete candidate OOF evaluation using the rule-based hierarchy described in the main Methods. Their absolute OOF performance is presented as post-selection descriptive internal validation, while the primary scientific comparisons are the within-algorithm paired history-minus-current contrasts.

1.2.10 Calibration, decision-curve analysis, and transportability

Calibration-in-the-large, calibration slope, Brier score, and probability calibration were evaluated within the nested validation design, with outer-test patients held aside from imputation, scaling, hyperparameter tuning, model fitting, and calibration. In each outer-training set, tuned hyperparameters were fixed and a separately seeded three-fold patient-level OOF procedure generated raw calibration probabilities. The calibrator used logistic regression of outcome on logit-clipped raw probability, with penalty=None, solver=lbfgs, max_iter=3000, and C=10^8 as a software-compatibility fallback. Probabilities were clipped to [10^-7, 1-10^-7]. The fitted calibrator was applied to raw outer-test probabilities from the model refitted on the complete outer-training set. Bootstrap intervals for the primary repeated-CV metrics summarize patient-level resampling variability around the realized OOF predictions. The separate

500-replicate model-development bootstrap repeated structural screening, preprocessing, tuning, fitting, and calibration and evaluated performance in out-of-bag patients. Its 2.5th-97.5th percentile ranges summarize development stability across bootstrap-developed models. Decision-curve analysis used averaged repeated-CV OOF probabilities as a supplementary assessment, with representative-model selection completed independently. Because the study was conducted at a single center with complete T0-T3 follow-up, temporal and multicenter validation will be important for assessing transportability. Future clinical translation should also consider recalibration where appropriate and prospective evaluation of clinical impact.

1.2.11 Reproducibility materials and data governance

Computational reproducibility is supported by the documented analysis pipeline, software versions, data schema, tuning specifications, and validation procedures. Patient-level clinical data, exact dates, OOF probabilities, fold assignments, and linked landmark records remain under controlled access in accordance with ethics and data-governance requirements. The manuscript and Supplementary Tables provide the parameter settings and procedures needed for independent implementation of the modeling workflow.

2 Supplementary Figures and Tables

2.1 Supplementary Tables

Supplementary Table 1. Predictor definitions and feature-set construction

Supplementary Table 1A. Feature-set rules

Feature set	Composition	Operational definition
landmark-only	Landmark indicator only	For pooled L1-L2, L1 is the reference and an L2 indicator represents landmark-specific baseline risk.
baseline	landmark-only + baseline-fixed predictors	Sex, baseline age, diabetes duration, and height. Prespecified landmark interactions are added where applicable.
current	baseline + current clinical information	Current landmark measurements plus the current status of each non-target comorbidity and a comorbidity count. Target-specific direct diagnostic proxies are excluded in the primary analysis.
history	current + longitudinal summaries	Adds prior mean and most recent change for prespecified history variables, using only information available up to the landmark.

Supplementary Table 1B. Predictor dictionary

Predictor/source	Unit or coding	Role in primary analysis	Feature-set inclusion	History summary	Transformation
Sex (0=female, 1=male)	binary	Baseline-fixed predictor	baseline, current, history	No	None
Age (years)	years	Baseline-fixed predictor	baseline, current, history	No	None
Diabetes duration (years)	years	Baseline-fixed predictor	baseline, current, history	No	None
Height (cm)	cm	Baseline-fixed predictor	baseline, current, history	No	None
Weight (kg)	kg	History-summary source only	history summaries only	Prior mean + recent change	None
Body mass index (kg/m²)	kg/m²	Current predictor and history-summary source	current, history	Prior mean + recent change	None
Waist circumference (cm)	cm	Current predictor and history-summary source	current, history	Prior mean + recent change	None
Hip circumference (cm)	cm	Raw repeated measure not used in primary candidate set	No	No	None
Waist-to-hip ratio	unitless	Current predictor and history-summary source	current, history	Prior mean + recent change	None
Fasting plasma glucose (mmol/L)	mmol/L	Current predictor and history-summary source	current, history	Prior mean + recent change	None
Glycated hemoglobin (%)	%	Current predictor and history-summary source	current, history	Prior mean + recent change	None
Systolic blood pressure (mmHg)	mm Hg	Current predictor and history-summary source	current, history	Prior mean + recent change	None
Diastolic blood pressure (mmHg)	mm Hg	Current predictor and history-summary source	current, history	Prior mean + recent change	None
Urinary albumin-to-creatinine ratio (mg/mmol)	mg/mmol	Current predictor and history-summary source	current, history	Prior mean + recent change	log(1+x)
Urinary creatinine	Source-recorded scale	Raw repeated measure not used in primary candidate set	No	No	None
Urinary microalbumin	Source-recorded scale	Raw repeated measure not used in primary candidate set	No	No	None
24-hour urinary total protein	Source-recorded scale	Current predictor	current, history	No	log(1+x)
Serum creatinine	µmol/L	Current predictor and history-summary source	current, history	Prior mean + recent change	None
Serum uric acid	µmol/L	Current predictor and history-summary source	current, history	Prior mean + recent change	None

Predictor/source	Unit or coding	Role in primary analysis	Feature-set inclusion	History summary	Transformation
Total cholesterol	mmol/L	Current predictor and history-summary source	current, history	Prior mean + recent change	None
Low-density lipoprotein cholesterol	mmol/L	Current predictor and history-summary source	current, history	Prior mean + recent change	None
High-density lipoprotein cholesterol	mmol/L	Current predictor and history-summary source	current, history	Prior mean + recent change	None
Triglycerides	mmol/L	Current predictor and history-summary source	current, history	Prior mean + recent change	log(1+x)
Alanine aminotransferase	U/L	Current predictor and history-summary source	current, history	Prior mean + recent change	log(1+x)
Aspartate aminotransferase	U/L	Current predictor and history-summary source	current, history	Prior mean + recent change	log(1+x)
Gamma-glutamyl transferase	U/L	Current predictor and history-summary source	current, history	Prior mean + recent change	log(1+x)
Total bilirubin	Source-recorded scale	Current predictor	current, history	No	None
Direct bilirubin	Source-recorded scale	Current predictor	current, history	No	None
Total protein	Source-recorded scale	Current predictor	current, history	No	None
Cardiac troponin I	Source-recorded scale	Current predictor	current, history	No	log(1+x)
Creatine kinase-MB	Source-recorded scale	Current predictor	current, history	No	log(1+x)
White blood cell count	$\times 10^9$/L	Current predictor	current, history	No	None
Resting electrocardiogram result	Binary/categorical, source recorded	Current predictor	current, history	No	None
Hepatic steatosis grade on abdominal ultrasonography	Ordinal, source recorded	Current predictor	current, history	No	None
Fundus examination result	Binary/categorical, source recorded	Current predictor	current, history	No	None
Current status of each non-target diabetes-related outcome	binary	Derived current comorbidity predictors	current, history	No	None
Count of non-target comorbidities	count	Derived current comorbidity burden	current, history	No	None
Landmark indicator	binary	Landmark-time predictor	all feature sets	No	None

Supplementary Table 1C. Prespecified landmark interactions

Variable	Interaction specification
Age (years)	Interaction with L2 indicator when the corresponding base feature is present
Diabetes duration (years)	Interaction with L2 indicator when the corresponding base feature is present
Body mass index (kg/m²)	Interaction with L2 indicator when the corresponding base feature is present
Glycated hemoglobin (%)	Interaction with L2 indicator when the corresponding base feature is present
Serum creatinine	Interaction with L2 indicator when the corresponding base feature is present
Low-density lipoprotein cholesterol	Interaction with L2 indicator when the corresponding base feature is present

Supplementary Table 1D. Outcome-specific direct diagnostic proxies

Target outcome	Variables excluded in the primary analysis
Hypertension	Systolic blood pressure (mmHg), Diastolic blood pressure (mmHg)
Coronary heart disease	Resting electrocardiogram result, Cardiac troponin I, Creatine kinase-MB
Diabetic kidney disease	Urinary albumin-to-creatinine ratio (mg/mmol), 24-hour urinary total protein

Target outcome	Variables excluded in the primary analysis
Diabetic retinopathy	Fundus examination result
Metabolic dysfunction-associated steatotic liver disease	Hepatic steatosis grade on abdominal ultrasonography

Note. These variables were measured at the landmark and were therefore temporally available. Because of their close relationship to the contemporaneous diagnostic process, the primary analysis omitted them from the candidate sets and evaluated them separately in sensitivity analyses. In Supplementary Table 1B, "source recorded" indicates that the source-recorded scale was retained when a specific unit was not encoded in the analysis variable dictionary.

Supplementary Table 2. Complete primary-analysis performance and paired comparisons

Supplementary Table 2A. Pooled L1-L2 OOF performance for all candidate combinations

Outcome	Feature set	Model	AUC (95% CI)	AP (95% CI)	Brier (95% CI)	CITL (95% CI)	Calibration slope (95% CI)
Hypertension	landmark-only	Elastic-net	0.514 (0.476, 0.550)	0.140 (0.117, 0.168)	0.117 (0.101, 0.134)	0.009 (-0.191, 0.190)	0.201 (-0.609, 1.003)
Hypertension	landmark-only	LightGBM	0.504 (0.466, 0.542)	0.139 (0.117, 0.168)	0.118 (0.102, 0.134)	0.003 (-0.186, 0.182)	0.019 (-0.754, 0.870)
Hypertension	baseline	Elastic-net	0.650 (0.594, 0.699)	0.239 (0.186, 0.312)	0.113 (0.098, 0.129)	0.001 (-0.206, 0.193)	0.951 (0.615, 1.273)
Hypertension	baseline	LightGBM	0.613 (0.558, 0.664)	0.204 (0.167, 0.260)	0.115 (0.099, 0.131)	0.001 (-0.192, 0.180)	0.841 (0.443, 1.222)
Hypertension	current	Elastic-net	0.693 (0.642, 0.743)	0.274 (0.216, 0.356)	0.110 (0.095, 0.125)	0.007 (-0.191, 0.187)	0.975 (0.680, 1.311)
Hypertension	current	LightGBM	0.667 (0.614, 0.713)	0.249 (0.201, 0.320)	0.112 (0.097, 0.127)	0.001 (-0.196, 0.192)	0.966 (0.644, 1.280)
Hypertension	history	Elastic-net	0.790 (0.748, 0.828)	0.343 (0.278, 0.423)	0.104 (0.089, 0.118)	-0.002 (-0.207, 0.185)	0.953 (0.755, 1.203)
Hypertension	history	LightGBM	0.785 (0.741, 0.825)	0.389 (0.321, 0.469)	0.100 (0.085, 0.115)	-0.017 (-0.227, 0.173)	1.006 (0.829, 1.209)
Coronary heart disease	landmark-only	Elastic-net	0.477 (0.461, 0.494)	0.167 (0.148, 0.192)	0.145 (0.131, 0.161)	0.006 (-0.154, 0.161)	-0.784 (-1.543, -0.122)
Coronary heart disease	landmark-only	LightGBM	0.475 (0.458, 0.492)	0.165 (0.145, 0.189)	0.145 (0.131, 0.160)	0.000 (-0.160, 0.145)	-1.499 (-2.294, -0.746)
Coronary heart disease	baseline	Elastic-net	0.697 (0.660, 0.736)	0.315 (0.263, 0.384)	0.135 (0.121, 0.148)	-0.002 (-0.158, 0.149)	0.976 (0.775, 1.225)
Coronary heart disease	baseline	LightGBM	0.711 (0.677, 0.745)	0.311 (0.268, 0.370)	0.131 (0.119, 0.144)	-0.005 (-0.163, 0.151)	0.984 (0.815, 1.183)
Coronary heart disease	current	Elastic-net	0.698 (0.657, 0.736)	0.341 (0.284, 0.408)	0.133 (0.120, 0.147)	0.001 (-0.171, 0.156)	0.973 (0.750, 1.221)
Coronary heart disease	current	LightGBM	0.722 (0.688, 0.755)	0.335 (0.289, 0.396)	0.132 (0.119, 0.146)	-0.011 (-0.158, 0.155)	1.008 (0.848, 1.195)
Coronary heart disease	history	Elastic-net	0.699 (0.658, 0.737)	0.337 (0.286, 0.397)	0.134 (0.121, 0.147)	-0.001 (-0.169, 0.156)	1.013 (0.788, 1.250)
Coronary heart disease	history	LightGBM	0.719 (0.688, 0.755)	0.332 (0.288, 0.391)	0.132 (0.120, 0.145)	-0.009 (-0.165, 0.144)	1.026 (0.866, 1.236)
Diabetic kidney disease	landmark-only	Elastic-net	0.485 (0.456, 0.515)	0.091 (0.077, 0.110)	0.085 (0.073, 0.099)	-0.011 (-0.200, 0.169)	-0.341 (-1.011, 0.334)
Diabetic kidney disease	landmark-only	LightGBM	0.493 (0.456, 0.527)	0.093 (0.076, 0.113)	0.085 (0.072, 0.099)	0.007 (-0.198, 0.188)	-0.278 (-1.263, 0.675)
Diabetic kidney disease	baseline	Elastic-net	0.762 (0.711, 0.809)	0.297 (0.227, 0.380)	0.076 (0.065, 0.087)	-0.003 (-0.229, 0.174)	0.994 (0.792, 1.222)
Diabetic kidney disease	baseline	LightGBM	0.751 (0.701, 0.795)	0.270 (0.211, 0.352)	0.077 (0.066, 0.089)	-0.011 (-0.222, 0.178)	0.989 (0.775, 1.209)
Diabetic kidney disease	current	Elastic-net	0.767 (0.720, 0.812)	0.264 (0.207, 0.344)	0.077 (0.066, 0.089)	-0.010 (-0.217, 0.191)	0.924 (0.738, 1.150)
Diabetic kidney disease	current	LightGBM	0.745 (0.698, 0.793)	0.240 (0.189, 0.315)	0.079 (0.068, 0.091)	-0.004 (-0.213, 0.197)	0.948 (0.747, 1.176)
Diabetic kidney disease	history	Elastic-net	0.757 (0.712, 0.800)	0.253 (0.196, 0.330)	0.078 (0.067, 0.090)	-0.017 (-0.220, 0.171)	0.910 (0.728, 1.121)
Diabetic kidney disease	history	LightGBM	0.744 (0.701, 0.789)	0.240 (0.191, 0.315)	0.079 (0.067, 0.091)	-0.007 (-0.231, 0.186)	0.961 (0.774, 1.178)
Diabetic retinopathy	landmark-only	Elastic-net	0.566 (0.514, 0.614)	0.093 (0.075, 0.116)	0.073 (0.061, 0.086)	0.000 (-0.226, 0.199)	0.929 (0.321, 1.560)
Diabetic retinopathy	landmark-only	LightGBM	0.566 (0.512, 0.616)	0.093 (0.076, 0.115)	0.073 (0.062, 0.086)	-0.004 (-0.217, 0.187)	0.961 (0.334, 1.617)
Diabetic retinopathy	baseline	Elastic-net	0.910 (0.888, 0.933)	0.487 (0.411, 0.576)	0.055 (0.046, 0.064)	-0.007 (-0.280, 0.238)	0.950 (0.830, 1.135)

Outcome	Feature set	Model	AUC (95% CI)	AP (95% CI)	Brier (95% CI)	CITL (95% CI)	Calibration slope (95% CI)
Diabetic retinopathy	baseline	LightGBM	0.909 (0.884, 0.931)	0.478 (0.396, 0.572)	0.054 (0.045, 0.064)	-0.005 (-0.266, 0.243)	0.981 (0.855, 1.140)
Diabetic retinopathy	current	Elastic-net	0.921 (0.899, 0.941)	0.566 (0.481, 0.652)	0.050 (0.041, 0.059)	-0.016 (-0.286, 0.239)	0.936 (0.815, 1.103)
Diabetic retinopathy	current	LightGBM	0.915 (0.893, 0.935)	0.517 (0.436, 0.608)	0.052 (0.044, 0.062)	-0.028 (-0.299, 0.225)	0.952 (0.832, 1.091)
Diabetic retinopathy	history	Elastic-net	0.920 (0.901, 0.939)	0.548 (0.473, 0.633)	0.051 (0.043, 0.060)	-0.015 (-0.281, 0.230)	0.931 (0.822, 1.081)
Diabetic retinopathy	history	LightGBM	0.915 (0.891, 0.936)	0.522 (0.435, 0.611)	0.052 (0.043, 0.061)	-0.021 (-0.300, 0.231)	0.947 (0.833, 1.089)
Metabolic dysfunction-associated steatotic liver disease	landmark-only	Elastic-net	0.615 (0.568, 0.660)	0.149 (0.117, 0.185)	0.100 (0.083, 0.119)	-0.002 (-0.254, 0.232)	1.034 (0.642, 1.566)
Metabolic dysfunction-associated steatotic liver disease	landmark-only	LightGBM	0.618 (0.575, 0.664)	0.149 (0.120, 0.186)	0.100 (0.083, 0.119)	0.003 (-0.242, 0.236)	0.858 (0.549, 1.297)
Metabolic dysfunction-associated steatotic liver disease	baseline	Elastic-net	0.690 (0.631, 0.746)	0.204 (0.158, 0.274)	0.099 (0.082, 0.117)	0.004 (-0.247, 0.226)	1.020 (0.638, 1.454)
Metabolic dysfunction-associated steatotic liver disease	baseline	LightGBM	0.675 (0.619, 0.728)	0.193 (0.154, 0.252)	0.100 (0.082, 0.117)	0.003 (-0.266, 0.230)	0.908 (0.583, 1.264)
Metabolic dysfunction-associated steatotic liver disease	current	Elastic-net	0.912 (0.880, 0.941)	0.626 (0.535, 0.737)	0.064 (0.051, 0.078)	-0.011 (-0.305, 0.261)	0.970 (0.732, 1.303)
Metabolic dysfunction-associated steatotic liver disease	current	LightGBM	0.927 (0.900, 0.951)	0.661 (0.565, 0.762)	0.061 (0.047, 0.075)	-0.014 (-0.360, 0.292)	0.934 (0.812, 1.105)
Metabolic dysfunction-associated steatotic liver disease	history	Elastic-net	0.934 (0.908, 0.957)	0.669 (0.565, 0.782)	0.059 (0.044, 0.073)	-0.015 (-0.339, 0.275)	0.958 (0.713, 1.344)
Metabolic dysfunction-associated steatotic liver disease	history	LightGBM	0.952 (0.933, 0.969)	0.740 (0.649, 0.825)	0.053 (0.041, 0.067)	0.010 (-0.351, 0.370)	0.960 (0.830, 1.142)

Note. Point estimates are averages of repeat-specific OOF metrics across the 10 outer-CV repeats. The 95% confidence intervals were estimated with 1,000 patient-level cluster bootstrap samples applied to the completed repeated-CV OOF prediction records.

Supplementary Table 2B. Paired history-versus-current differences

Outcome	Model	ΔAUC (95% CI)	ΔAP (95% CI)	ΔBrier (95% CI)
Hypertension	Elastic-net	0.097 (0.057, 0.136)	0.069 (0.027, 0.118)	-0.006 (-0.012, -0.002)
Hypertension	LightGBM	0.118 (0.081, 0.158)	0.140 (0.090, 0.192)	-0.012 (-0.018, -0.007)
Coronary heart disease	Elastic-net	0.001 (-0.011, 0.013)	-0.004 (-0.027, 0.012)	0.000 (-0.001, 0.002)
Coronary heart disease	LightGBM	-0.003 (-0.012, 0.008)	-0.002 (-0.018, 0.014)	0.000 (-0.001, 0.001)
Diabetic kidney disease	Elastic-net	-0.010 (-0.021, 0.001)	-0.011 (-0.031, 0.008)	0.001 (0.000, 0.002)
Diabetic kidney disease	LightGBM	-0.001 (-0.015, 0.013)	0.000 (-0.021, 0.019)	0.000 (-0.001, 0.001)
Diabetic retinopathy	Elastic-net	-0.001 (-0.007, 0.005)	-0.018 (-0.044, 0.010)	0.001 (-0.001, 0.003)
Diabetic retinopathy	LightGBM	-0.001 (-0.007, 0.007)	0.005 (-0.019, 0.024)	0.000 (-0.002, 0.001)
Metabolic dysfunction-associated steatotic liver disease	Elastic-net	0.022 (0.002, 0.042)	0.043 (-0.003, 0.094)	-0.005 (-0.010, 0.000)
Metabolic dysfunction-associated steatotic liver disease	LightGBM	0.025 (0.009, 0.041)	0.079 (0.030, 0.121)	-0.008 (-0.013, -0.002)

Note. Candidate A denotes the current feature set and candidate B denotes the history feature set. Reported differences represent history minus current.

Supplementary Table 2C. Paired LightGBM-versus-elastic-net differences

Outcome	Feature set	ΔAUC (95% CI)	ΔAP (95% CI)	ΔBrier (95% CI)
Hypertension	landmark-only	-0.009 (-0.021, 0.003)	-0.001 (-0.006, 0.004)	0.000 (0.000, 0.000)
Hypertension	baseline	-0.037 (-0.054, -0.020)	-0.035 (-0.063, -0.011)	0.002 (0.001, 0.003)
Hypertension	current	-0.026 (-0.047, -0.003)	-0.024 (-0.056, 0.004)	0.002 (-0.001, 0.004)
Hypertension	history	-0.004 (-0.030, 0.019)	0.046 (-0.003, 0.088)	-0.004 (-0.008, 0.000)
Coronary heart disease	landmark-only	-0.002 (-0.013, 0.008)	-0.002 (-0.007, 0.003)	0.000 (0.000, 0.000)
Coronary heart disease	baseline	0.014 (-0.007, 0.035)	-0.004 (-0.038, 0.025)	-0.003 (-0.006, -0.001)
Coronary heart disease	current	0.024 (0.001, 0.048)	-0.006 (-0.039, 0.026)	-0.001 (-0.004, 0.002)
Coronary heart disease	history	0.020 (-0.003, 0.042)	-0.004 (-0.031, 0.027)	-0.001 (-0.004, 0.001)
Diabetic kidney disease	landmark-only	0.008 (-0.006, 0.020)	0.002 (-0.001, 0.005)	0.000 (0.000, 0.000)
Diabetic kidney disease	baseline	-0.011 (-0.030, 0.007)	-0.027 (-0.058, 0.003)	0.001 (0.000, 0.003)
Diabetic kidney disease	current	-0.022 (-0.041, -0.003)	-0.024 (-0.058, 0.004)	0.001 (0.000, 0.003)
Diabetic kidney disease	history	-0.013 (-0.033, 0.009)	-0.013 (-0.049, 0.022)	0.001 (-0.001, 0.003)
Diabetic retinopathy	landmark-only	0.000 (-0.005, 0.005)	0.000 (-0.002, 0.002)	0.000 (0.000, 0.000)
Diabetic retinopathy	baseline	-0.001 (-0.010, 0.008)	-0.010 (-0.045, 0.033)	0.000 (-0.003, 0.002)
Diabetic retinopathy	current	-0.006 (-0.016, 0.006)	-0.049 (-0.079, -0.009)	0.002 (0.000, 0.005)
Diabetic retinopathy	history	-0.006 (-0.018, 0.007)	-0.026 (-0.061, 0.012)	0.001 (-0.002, 0.004)
Metabolic dysfunction-associated steatotic liver disease	landmark-only	0.002 (-0.005, 0.008)	0.000 (-0.003, 0.004)	0.000 (0.000, 0.000)
Metabolic dysfunction-associated steatotic liver disease	baseline	-0.015 (-0.039, 0.011)	-0.011 (-0.041, 0.014)	0.001 (0.000, 0.002)
Metabolic dysfunction-associated steatotic liver disease	current	0.015 (-0.003, 0.034)	0.035 (-0.032, 0.090)	-0.003 (-0.010, 0.003)
Metabolic dysfunction-associated steatotic liver disease	history	0.018 (0.002, 0.034)	0.071 (-0.005, 0.129)	-0.006 (-0.013, 0.001)

Note. Candidate A denotes elastic-net and candidate B denotes LightGBM. Reported differences represent LightGBM minus elastic-net.

Supplementary Table 3. Model interpretation and bootstrap stability

Outcome	Feature set	Model	Model role	Rank	Feature	Importance/coefficient	Bootstrap frequency (top-k or selection)	Secondary stability measure	Quantity
Hypertension	history	LightGBM	Representative model	1	Prior mean of Triglycerides, log(1+x) transformed	0.443	1.00	1.0	Mean |SHAP|
Hypertension	history	LightGBM	Representative model	2	Prior mean of Serum uric acid	0.231	0.97	4.0	Mean |SHAP|
Hypertension	history	LightGBM	Representative model	3	Most recent change in Serum creatinine	0.227	0.84	5.0	Mean |SHAP|
Hypertension	history	LightGBM	Representative model	4	Baseline Age (years)	0.202	0.95	4.0	Mean |SHAP|
Hypertension	history	LightGBM	Representative model	5	Prior mean of Serum creatinine	0.179	0.87	5.0	Mean |SHAP|
Hypertension	history	LightGBM	Representative model	6	Current White blood cell count	0.142	0.60	9.0	Mean |SHAP|
Hypertension	history	LightGBM	Representative model	7	Most recent change in Serum uric acid	0.115	0.56	9.0	Mean |SHAP|
Hypertension	history	LightGBM	Representative model	8	Prior mean of High-density lipoprotein cholesterol	0.115	0.58	9.0	Mean |SHAP|
Hypertension	history	LightGBM	Representative model	9	Most recent change in Low-density lipoprotein cholesterol	0.105	0.54	10.0	Mean |SHAP|
Hypertension	history	LightGBM	Representative model	10	Most recent change in Body mass index (kg/m²)	0.104	0.25	15.5	Mean |SHAP|
Hypertension	history	Elastic-net	Elastic-net comparison model	1	Baseline Age (years)	0.603	1.00	1.00	Scaled coefficient
Hypertension	history	Elastic-net	Elastic-net comparison model	2	Prior mean of Triglycerides, log(1+x) transformed	0.543	1.00	1.00	Scaled coefficient
Hypertension	history	Elastic-net	Elastic-net comparison model	3	Prior mean of Serum uric acid	0.480	1.00	1.00	Scaled coefficient
Hypertension	history	Elastic-net	Elastic-net comparison model	4	Current White blood cell count	0.313	0.99	1.00	Scaled coefficient
Hypertension	history	Elastic-net	Elastic-net comparison model	5	Prior mean of High-density lipoprotein cholesterol	-0.218	0.96	1.00	Scaled coefficient
Hypertension	history	Elastic-net	Elastic-net comparison model	6	Interaction with L2, Glycated hemoglobin (%)	-0.217	0.72	1.00	Scaled coefficient
Hypertension	history	Elastic-net	Elastic-net comparison model	7	Current Hepatic steatosis grade on abdominal ultrasonography	0.185	0.62	1.00	Scaled coefficient
Hypertension	history	Elastic-net	Elastic-net comparison model	8	Most recent change in Total cholesterol	-0.180	0.98	1.00	Scaled coefficient
Hypertension	history	Elastic-net	Elastic-net comparison model	9	Prior mean of Serum creatinine	0.154	0.83	1.00	Scaled coefficient
Hypertension	history	Elastic-net	Elastic-net comparison model	10	Current Direct bilirubin	0.138	0.80	1.00	Scaled coefficient
Coronary heart disease	current	LightGBM	Representative model	1	Baseline Diabetes duration (years)	0.635	1.00	1.0	Mean |SHAP|
Coronary heart disease	current	LightGBM	Representative model	2	Baseline Age (years)	0.287	1.00	2.0	Mean |SHAP|
Coronary heart disease	current	LightGBM	Representative model	3	Current Serum creatinine	0.135	0.95	4.0	Mean |SHAP|
Coronary heart disease	current	LightGBM	Representative model	4	Current Total cholesterol	0.116	0.85	5.5	Mean |SHAP|
Coronary heart disease	current	LightGBM	Representative model	5	Current Low-density lipoprotein cholesterol	0.095	0.60	9.0	Mean |SHAP|
Coronary heart disease	current	LightGBM	Representative model	6	Current 24-hour urinary total protein, log(1+x) transformed	0.072	0.39	13.5	Mean |SHAP|
Coronary heart disease	current	LightGBM	Representative model	7	Current Urinary albumin-to-creatinine ratio (mg/mmol), log(1+x) transformed	0.062	0.45	12.0	Mean |SHAP|
Coronary heart disease	current	LightGBM	Representative model	8	Current Alanine aminotransferase, log(1+x) transformed	0.059	0.53	10.0	Mean |SHAP|

Outcome	Feature set	Model	Model role	Rank	Feature	Importance/coefficient	Bootstrap frequency (top-k or selection)	Secondary stability measure	Quantity
Coronary heart disease	current	LightGBM	Representative model	9	Current Total bilirubin	0.052	0.38	12.0	Mean |SHAP|
Coronary heart disease	current	LightGBM	Representative model	10	Current White blood cell count	0.045	0.32	14.5	Mean |SHAP|
Coronary heart disease	current	Elastic-net	Elastic-net comparison model	1	Baseline Age (years)	0.578	1.00	1.00	Scaled coefficient
Coronary heart disease	current	Elastic-net	Elastic-net comparison model	2	Baseline Diabetes duration (years)	0.497	1.00	1.00	Scaled coefficient
Coronary heart disease	current	Elastic-net	Elastic-net comparison model	3	Current Serum creatinine	0.257	0.98	1.00	Scaled coefficient
Coronary heart disease	current	Elastic-net	Elastic-net comparison model	4	Current 24-hour urinary total protein, log(1+x) transformed	-0.225	1.00	1.00	Scaled coefficient
Coronary heart disease	current	Elastic-net	Elastic-net comparison model	5	Current Urinary albumin-to-creatinine ratio (mg/mmol), log(1+x) transformed	0.196	0.99	1.00	Scaled coefficient
Coronary heart disease	current	Elastic-net	Elastic-net comparison model	6	Current Total cholesterol	-0.119	0.93	1.00	Scaled coefficient
Coronary heart disease	current	Elastic-net	Elastic-net comparison model	7	Current non-target comorbidity, Hypertension	0.106	0.68	1.00	Scaled coefficient
Coronary heart disease	current	Elastic-net	Elastic-net comparison model	8	Current Alanine aminotransferase, log(1+x) transformed	-0.097	0.70	1.00	Scaled coefficient
Coronary heart disease	current	Elastic-net	Elastic-net comparison model	9	Current Low-density lipoprotein cholesterol	0.052	0.51	0.94	Scaled coefficient
Coronary heart disease	current	Elastic-net	Elastic-net comparison model	10	Current High-density lipoprotein cholesterol	-0.051	0.67	0.96	Scaled coefficient
Diabetic kidney disease	baseline	Elastic-net	Representative model	1	Baseline Diabetes duration (years)	1.088	1.00	1.00	Scaled coefficient
Diabetic kidney disease	baseline	Elastic-net	Representative model	2	Baseline Age (years)	0.820	1.00	1.00	Scaled coefficient
Diabetic kidney disease	baseline	Elastic-net	Representative model	3	Interaction with L2, Diabetes duration (years)	-0.175	0.96	1.00	Scaled coefficient
Diabetic retinopathy	current	Elastic-net	Representative model	1	Baseline Age (years)	1.470	1.00	1.00	Scaled coefficient
Diabetic retinopathy	current	Elastic-net	Representative model	2	Baseline Diabetes duration (years)	1.163	1.00	1.00	Scaled coefficient
Diabetic retinopathy	current	Elastic-net	Representative model	3	Interaction with L2, Diabetes duration (years)	0.460	1.00	1.00	Scaled coefficient
Diabetic retinopathy	current	Elastic-net	Representative model	4	Current Urinary albumin-to-creatinine ratio (mg/mmol), log(1+x) transformed	0.355	1.00	1.00	Scaled coefficient
Diabetic retinopathy	current	Elastic-net	Representative model	5	Other-comorbidity count, Other comorbidities	0.274	0.96	1.00	Scaled coefficient
Diabetic retinopathy	current	Elastic-net	Representative model	6	Current Serum uric acid	-0.189	0.91	1.00	Scaled coefficient
Diabetic retinopathy	current	Elastic-net	Representative model	7	Current Gamma-glutamyl transferase, log(1+x) transformed	0.104	0.68	0.97	Scaled coefficient
Diabetic retinopathy	current	Elastic-net	Representative model	8	Current Body mass index (kg/m²)	-0.101	0.63	0.97	Scaled coefficient
Diabetic retinopathy	current	Elastic-net	Representative model	9	Current Total bilirubin	-0.070	0.67	0.97	Scaled coefficient

Outcome	Feature set	Model	Model role	Rank	Feature	Importance/coefficient	Bootstrap frequency (top-k or selection)	Secondary stability measure	Quantity
Diabetic retinopathy	current	Elastic-net	Representative model	10	Current Alanine aminotransferase, log(1+x) transformed	0.066	0.46	0.98	Scaled coefficient
Metabolic dysfunction-associated steatotic liver disease	history	LightGBM	Representative model	1	Prior mean of Body mass index (kg/m²)	1.310	1.00	1.0	Mean |SHAP|
Metabolic dysfunction-associated steatotic liver disease	history	LightGBM	Representative model	2	Current Body mass index (kg/m²)	0.597	1.00	2.0	Mean |SHAP|
Metabolic dysfunction-associated steatotic liver disease	history	LightGBM	Representative model	3	Most recent change in Total cholesterol	0.268	0.85	4.0	Mean |SHAP|
Metabolic dysfunction-associated steatotic liver disease	history	LightGBM	Representative model	4	Current High-density lipoprotein cholesterol	0.246	0.89	5.0	Mean |SHAP|
Metabolic dysfunction-associated steatotic liver disease	history	LightGBM	Representative model	5	Prior mean of Aspartate aminotransferase, log(1+x) transformed	0.187	0.73	8.0	Mean |SHAP|
Metabolic dysfunction-associated steatotic liver disease	history	LightGBM	Representative model	6	Current Total cholesterol	0.154	0.65	8.5	Mean |SHAP|
Metabolic dysfunction-associated steatotic liver disease	history	LightGBM	Representative model	7	Most recent change in Serum uric acid	0.134	0.39	13.0	Mean |SHAP|
Metabolic dysfunction-associated steatotic liver disease	history	LightGBM	Representative model	8	Most recent change in Alanine aminotransferase, log(1+x) transformed	0.102	0.50	10.5	Mean |SHAP|
Metabolic dysfunction-associated steatotic liver disease	history	LightGBM	Representative model	9	Most recent change in Waist-to-hip ratio	0.094	0.21	19.0	Mean |SHAP|
Metabolic dysfunction-associated steatotic liver disease	history	LightGBM	Representative model	10	Current 24-hour urinary total protein, log(1+x) transformed	0.093	0.26	17.5	Mean |SHAP|
Metabolic dysfunction-associated steatotic liver disease	history	Elastic-net	Elastic-net comparison model	1	Prior mean of Body mass index (kg/m²)	1.181	1.00	1.00	Scaled coefficient
Metabolic dysfunction-associated steatotic liver disease	history	Elastic-net	Elastic-net comparison model	2	Current Body mass index (kg/m²)	0.725	1.00	1.00	Scaled coefficient
Metabolic dysfunction-associated steatotic liver disease	history	Elastic-net	Elastic-net comparison model	3	Prior mean of Waist circumference (cm)	0.293	0.91	1.00	Scaled coefficient
Metabolic dysfunction-associated steatotic liver disease	history	Elastic-net	Elastic-net comparison model	4	Most recent change in Alanine aminotransferase, log(1+x) transformed	-0.168	0.87	1.00	Scaled coefficient
Metabolic dysfunction-associated steatotic liver disease	history	Elastic-net	Elastic-net comparison model	5	Interaction with L2, Glycated hemoglobin (%)	-0.159	0.74	1.00	Scaled coefficient
Metabolic dysfunction-associated steatotic liver disease	history	Elastic-net	Elastic-net comparison model	6	Most recent change in Total cholesterol	-0.121	0.96	1.00	Scaled coefficient
Metabolic dysfunction-associated steatotic liver disease	history	Elastic-net	Elastic-net comparison model	7	Current High-density lipoprotein cholesterol	-0.118	0.69	1.00	Scaled coefficient
Metabolic dysfunction-associated steatotic liver disease	history	Elastic-net	Elastic-net comparison model	8	Interaction with L2, Body mass index (kg/m²)	-0.094	0.49	1.00	Scaled coefficient
Metabolic dysfunction-associated steatotic liver disease	history	Elastic-net	Elastic-net comparison model	9	Most recent change in Gamma-glutamyl transferase, log(1+x) transformed	0.083	0.95	0.99	Scaled coefficient
Metabolic dysfunction-associated steatotic liver disease	history	Elastic-net	Elastic-net comparison model	10	Most recent change in Waist-to-hip ratio	0.071	0.52	1.00	Scaled coefficient

Note. For LightGBM, the table reports the 10 highest global mean absolute SHAP values together with bootstrap top-k frequency and median rank. For elastic-net, it reports up to the 10 largest nonzero scaled coefficients together with bootstrap selection frequency and sign consistency among selected refits. Interpretation stability was assessed using 100 patient-level bootstrap refits. These summaries describe model-specific predictive contributions rather than causal effects.

Supplementary Table 4. Detailed sensitivity analyses

Supplementary Table 4A. History-versus-current sensitivity results

Scenario	Outcome	Main algorithm	ΔAUC history-current (95% CI)	ΔAP history-current (95% CI)	ΔBrier history-current (95% CI)
Primary analysis	Hypertension	LightGBM	0.118 (0.081, 0.158)	0.140 (0.090, 0.192)	-0.012 (-0.018, -0.007)
Primary analysis	Coronary heart disease	LightGBM	-0.003 (-0.012, 0.008)	-0.002 (-0.018, 0.014)	0.000 (-0.001, 0.001)
Primary analysis	Diabetic kidney disease	Elastic-net	-0.010 (-0.021, 0.001)	-0.011 (-0.031, 0.008)	0.001 (0.000, 0.002)
Primary analysis	Diabetic retinopathy	Elastic-net	-0.001 (-0.007, 0.005)	-0.018 (-0.044, 0.010)	0.001 (-0.001, 0.003)
Primary analysis	Metabolic dysfunction-associated steatotic liver disease	LightGBM	0.025 (0.009, 0.041)	0.079 (0.030, 0.121)	-0.008 (-0.013, -0.002)
As-observed outcome records	Hypertension	LightGBM	0.115 (0.075, 0.154)	0.131 (0.077, 0.187)	-0.012 (-0.017, -0.007)
As-observed outcome records	Coronary heart disease	LightGBM	-0.007 (-0.018, 0.004)	-0.003 (-0.020, 0.015)	0.000 (-0.001, 0.002)
As-observed outcome records	Diabetic kidney disease	Elastic-net	-0.006 (-0.017, 0.005)	-0.001 (-0.019, 0.018)	0.000 (-0.001, 0.001)
As-observed outcome records	Diabetic retinopathy	Elastic-net	-0.002 (-0.009, 0.005)	-0.023 (-0.052, 0.007)	0.001 (-0.001, 0.004)
As-observed outcome records	Metabolic dysfunction-associated steatotic liver disease	LightGBM	0.025 (0.008, 0.043)	0.067 (0.018, 0.113)	-0.007 (-0.012, -0.001)
Direct diagnostic proxies included	Hypertension	LightGBM	0.075 (0.045, 0.104)	0.113 (0.058, 0.168)	-0.012 (-0.017, -0.006)
Direct diagnostic proxies included	Coronary heart disease	LightGBM	-0.002 (-0.005, 0.001)	-0.013 (-0.028, 0.003)	0.000 (0.000, 0.001)
Direct diagnostic proxies included	Diabetic kidney disease	Elastic-net	0.001 (-0.006, 0.008)	0.008 (-0.047, 0.051)	-0.001 (-0.005, 0.003)
Direct diagnostic proxies included	Diabetic retinopathy	Elastic-net	-0.001 (-0.008, 0.005)	-0.023 (-0.052, 0.008)	0.001 (-0.001, 0.004)
Direct diagnostic proxies included	Metabolic dysfunction-associated steatotic liver disease	LightGBM	0.025 (0.008, 0.043)	0.067 (0.018, 0.113)	-0.007 (-0.012, -0.001)
Other comorbidity predictors removed	Hypertension	LightGBM	0.112 (0.073, 0.152)	0.133 (0.076, 0.189)	-0.012 (-0.017, -0.007)
Other comorbidity predictors removed	Coronary heart disease	LightGBM	-0.006 (-0.018, 0.005)	-0.005 (-0.025, 0.015)	0.000 (-0.001, 0.002)
Other comorbidity predictors removed	Diabetic kidney disease	Elastic-net	-0.005 (-0.018, 0.008)	-0.007 (-0.028, 0.013)	0.001 (-0.001, 0.002)
Other comorbidity predictors removed	Diabetic retinopathy	Elastic-net	-0.002 (-0.009, 0.004)	-0.022 (-0.053, 0.009)	0.001 (-0.001, 0.004)
Other comorbidity predictors removed	Metabolic dysfunction-associated steatotic liver disease	LightGBM	0.026 (0.008, 0.046)	0.082 (0.030, 0.128)	-0.008 (-0.013, -0.002)
Non-monotonic records excluded	Hypertension	LightGBM	0.115 (0.075, 0.154)	0.131 (0.077, 0.187)	-0.012 (-0.017, -0.007)
Non-monotonic records excluded	Coronary heart disease	LightGBM	-0.007 (-0.018, 0.004)	-0.003 (-0.020, 0.015)	0.000 (-0.001, 0.002)
Non-monotonic records excluded	Diabetic kidney disease	Elastic-net	-0.006 (-0.017, 0.005)	-0.001 (-0.019, 0.018)	0.000 (-0.001, 0.001)
Non-monotonic records excluded	Diabetic retinopathy	Elastic-net	-0.002 (-0.009, 0.005)	-0.023 (-0.052, 0.007)	0.001 (-0.001, 0.004)
Non-monotonic records excluded	Metabolic dysfunction-associated steatotic liver disease	LightGBM	0.029 (0.011, 0.050)	0.088 (0.035, 0.138)	-0.009 (-0.015, -0.002)
Pooled L0-L2	Hypertension	LightGBM	0.031 (0.014, 0.047)	0.036 (0.015, 0.058)	-0.004 (-0.007, -0.002)
Pooled L0-L2	Coronary heart disease	LightGBM	-0.013 (-0.023, -0.005)	-0.029 (-0.053, -0.007)	0.001 (0.000, 0.003)
Pooled L0-L2	Diabetic kidney disease	Elastic-net	-0.008 (-0.023, 0.008)	-0.042 (-0.067, -0.012)	0.002 (0.000, 0.003)
Pooled L0-L2	Diabetic retinopathy	Elastic-net	-0.014 (-0.025, -0.003)	-0.061 (-0.106, -0.017)	0.002 (0.000, 0.005)
Pooled L0-L2	Metabolic dysfunction-associated steatotic liver disease	LightGBM	0.005 (0.001, 0.008)	0.000 (-0.009, 0.008)	-0.002 (-0.003, 0.000)

Supplementary Table 4B. Algorithm sensitivity results for configurations discussed in the main text

Scenario	Outcome	Feature set	ΔAUC LightGBM-Elastic-net (95% CI)	ΔAP LightGBM-Elastic-net (95% CI)	ΔBrier LightGBM-Elastic-net (95% CI)
Primary analysis	Coronary heart disease	current	0.024 (0.001, 0.048)	-0.006 (-0.039, 0.026)	-0.001 (-0.004, 0.002)
Primary analysis	Diabetic retinopathy	current	-0.006 (-0.016, 0.006)	-0.049 (-0.079, -0.009)	0.002 (0.000, 0.005)

Scenario	Outcome	Feature set	ΔAUC LightGBM-Elastic-net (95% CI)	ΔAP LightGBM-Elastic-net (95% CI)	ΔBrier LightGBM-Elastic-net (95% CI)
Primary analysis	Metabolic dysfunction-associated steatotic liver disease	history	0.018 (0.002, 0.034)	0.071 (-0.005, 0.129)	-0.006 (-0.013, 0.001)
As-observed outcome records	Coronary heart disease	current	0.023 (-0.001, 0.049)	-0.010 (-0.046, 0.023)	-0.001 (-0.004, 0.002)
As-observed outcome records	Diabetic retinopathy	current	-0.007 (-0.019, 0.005)	-0.049 (-0.077, -0.011)	0.002 (0.000, 0.005)
As-observed outcome records	Metabolic dysfunction-associated steatotic liver disease	history	0.016 (0.000, 0.031)	0.058 (-0.021, 0.117)	-0.006 (-0.013, 0.001)
Direct diagnostic proxies included	Coronary heart disease	current	0.003 (0.000, 0.008)	0.004 (-0.013, 0.019)	0.000 (-0.001, 0.001)
Direct diagnostic proxies included	Diabetic retinopathy	current	-0.008 (-0.019, 0.005)	-0.056 (-0.085, -0.018)	0.003 (0.000, 0.005)
Direct diagnostic proxies included	Metabolic dysfunction-associated steatotic liver disease	history	0.008 (-0.007, 0.024)	0.009 (-0.071, 0.069)	0.000 (-0.008, 0.007)
Other comorbidity predictors removed	Coronary heart disease	current	0.023 (-0.001, 0.048)	-0.008 (-0.046, 0.029)	-0.001 (-0.004, 0.002)
Other comorbidity predictors removed	Diabetic retinopathy	current	-0.005 (-0.015, 0.007)	-0.038 (-0.070, 0.002)	0.002 (0.000, 0.004)
Other comorbidity predictors removed	Metabolic dysfunction-associated steatotic liver disease	history	0.018 (0.003, 0.034)	0.073 (-0.002, 0.130)	-0.007 (-0.014, 0.000)
Non-monotonic records excluded	Coronary heart disease	current	0.023 (-0.001, 0.049)	-0.010 (-0.046, 0.023)	-0.001 (-0.004, 0.002)
Non-monotonic records excluded	Diabetic retinopathy	current	-0.007 (-0.019, 0.005)	-0.049 (-0.077, -0.011)	0.002 (0.000, 0.005)
Non-monotonic records excluded	Metabolic dysfunction-associated steatotic liver disease	history	0.026 (0.012, 0.046)	0.081 (0.021, 0.137)	-0.008 (-0.016, -0.002)
Pooled L0-L2	Coronary heart disease	current	0.021 (0.005, 0.035)	0.008 (-0.029, 0.042)	-0.001 (-0.004, 0.001)
Pooled L0-L2	Diabetic retinopathy	current	-0.005 (-0.018, 0.006)	-0.043 (-0.074, -0.004)	0.001 (0.000, 0.003)
Pooled L0-L2	Metabolic dysfunction-associated steatotic liver disease	history	0.019 (0.008, 0.029)	-0.007 (-0.042, 0.030)	-0.016 (-0.022, -0.010)

Supplementary Table 4C. Effect of reintroducing contemporaneous direct diagnostic proxies

Outcome	Feature set	Model	Primary AUC	AUC with proxies	Primary AP	AP with proxies	Primary Brier	Brier with proxies
Coronary heart disease	current	LightGBM	0.722 (0.688, 0.755)	0.974 (0.963, 0.984)	0.335 (0.289, 0.396)	0.880 (0.844, 0.920)	0.132 (0.119, 0.146)	0.029 (0.021, 0.038)
Diabetic kidney disease	current	Elastic-net	0.767 (0.720, 0.812)	0.972 (0.956, 0.984)	0.264 (0.207, 0.344)	0.785 (0.734, 0.856)	0.077 (0.066, 0.089)	0.033 (0.025, 0.041)

Note. These variables were measured at the landmark and therefore did not represent future information. Their reintroduction was used to examine how predictors closely related to the contemporaneous diagnostic process affected apparent predictive performance.

Supplementary Table 5. Data-quality, OOF-integrity, and reproducibility audit

Supplementary Table 5A. Missingness across the 31 repeated measures

Visit	Median cleaned missingness	Maximum cleaned missingness
T0	6.2%	11.1%
T1	6.2%	9.6%
T2	6.2%	12.4%
T3	6.3%	10.8%

Supplementary Table 5B. Outcome-record audit

Outcome	Non-monotonic patient records	First recorded events T1-T2	First recorded events T2-T3
Hypertension	0	82	43

Outcome	Non-monotonic patient records	First recorded events T1-T2	First recorded events T2-T3
Coronary heart disease	1	123	78
Diabetic kidney disease	0	71	42
Diabetic retinopathy	0	34	66
Metabolic dysfunction-associated steatotic liver disease	8	65	15

Supplementary Table 5C. Primary-analysis OOF integrity checks

Audit check	Status	Detail
Required columns present	Passed	All required columns present
No duplicate prediction keys	Passed	No duplicates
Binary target coding	Passed	0 and 1
Probabilities finite and within 0-1	Passed	Raw and calibrated probabilities
Expected task grid complete	Passed	400/400 tasks passed
Expected repeat count	Passed	Expected 10; observed 10
Expected outer-fold count	Passed	400/400 tasks passed
One prediction per patient-landmark-repeat	Passed	Maximum count = 1
Paired risk sets identical across candidates	Passed	No outcome-repeat mismatches
Risk sets identical across repeats	Passed	No task mismatches

Supplementary Table 5D. Reproducibility settings

Item	Recorded value
Pipeline version	6.0-submission-final
Random seed	20260720
Outer cross-validation	5 folds x 10 repeats
Inner cross-validation	3 folds
Calibration OOF splitting	Separately seeded 3-fold patient-level splits within each outer-training set, using the selected hyperparameters
Sigmoid calibrator	Logistic regression on logit-clipped raw OOF probabilities; penalty=None (C=10^8 compatibility fallback), solver=lbfgs, max_iter=3000, clipping epsilon=10^-7
Calibration application	Outer-fold-specific calibrator applied to raw probabilities from the final model refit on the complete outer-training set; outer-test data excluded
Primary-analysis Optuna trials	30
Sensitivity-analysis outer CV repeats	5
Sensitivity-analysis Optuna trials	15
Primary landmarks	L1 and L2
History profile	Extended longitudinal history profile (extended_a)
History summaries	Recent change (recent_delta) and prior mean (prior_mean)
Direct proxies in primary analysis	Excluded
Other comorbidity predictors in primary analysis	Included

Item	Recorded value
Python	3.9.25
pandas	1.5.3
NumPy	1.23.5
SciPy	1.10.1
scikit-learn	1.5.2
LightGBM	3.3.2
Optuna	3.2.0

2.2 Supplementary Tables continued

Supplementary Table 6A. Full-development-data refit parameters

These parameter settings were obtained by retuning on the complete development data after internal validation. They supported the prespecified coefficient or SHAP interpretation analyses and the corresponding stability refits. Outer-fold OOF performance estimates were generated separately within the nested validation procedure.

Outcome	Feature set	Model	Role	Parameter	Full-data refit value	Source
HTN	history	lightgbm	primary	n_estimators	357	retune
HTN	history	lightgbm	primary	learning_rate	0.010060665319661823	retune
HTN	history	lightgbm	primary	num_leaves	12	retune
HTN	history	lightgbm	primary	max_depth	4	retune
HTN	history	lightgbm	primary	min_child_samples	73	retune
HTN	history	lightgbm	primary	subsample	0.9948955394131771	retune
HTN	history	lightgbm	primary	colsample_bytree	0.7976994760316838	retune
HTN	history	lightgbm	primary	reg_alpha	0.0011053541334079676	retune
HTN	history	lightgbm	primary	reg_lambda	0.1007584063699637	retune
HTN	history	lightgbm	primary	min_split_gain	0.8495259009569013	retune
HTN	history	lightgbm	primary	subsample_freq	1	retune
HTN	history	elasticnet	interpretable_secondary	C	0.10731574270312205	retune
HTN	history	elasticnet	interpretable_secondary	l1_ratio	0.6184738179957022	retune
CHD	current	lightgbm	primary	n_estimators	276	retune
CHD	current	lightgbm	primary	learning_rate	0.012561627677718163	retune
CHD	current	lightgbm	primary	num_leaves	7	retune
CHD	current	lightgbm	primary	max_depth	5	retune
CHD	current	lightgbm	primary	min_child_samples	96	retune
CHD	current	lightgbm	primary	subsample	0.9599170771582114	retune
CHD	current	lightgbm	primary	colsample_bytree	0.7395179866068865	retune
CHD	current	lightgbm	primary	reg_alpha	0.0010352627953476939	retune
CHD	current	lightgbm	primary	reg_lambda	1.4102301164985318	retune
CHD	current	lightgbm	primary	min_split_gain	0.6186010652035842	retune
CHD	current	lightgbm	primary	subsample_freq	1	retune
CHD	current	elasticnet	interpretable_secondary	C	0.1466799582473823	retune
CHD	current	elasticnet	interpretable_secondary	l1_ratio	0.9962700379028572	retune
DKD	baseline	elasticnet	primary	C	0.2542097370596191	retune
DKD	baseline	elasticnet	primary	l1_ratio	0.8450349126226349	retune
DR	current	elasticnet	primary	C	0.16823216333953322	retune
DR	current	elasticnet	primary	l1_ratio	0.8430492540272014	retune
MASLD	history	lightgbm	primary	n_estimators	154	retune
MASLD	history	lightgbm	primary	learning_rate	0.04925202530584622	retune
MASLD	history	lightgbm	primary	num_leaves	13	retune
MASLD	history	lightgbm	primary	max_depth	4	retune
MASLD	history	lightgbm	primary	min_child_samples	73	retune
MASLD	history	lightgbm	primary	subsample	0.915657449139295	retune
MASLD	history	lightgbm	primary	colsample_bytree	0.7110794057541349	retune
MASLD	history	lightgbm	primary	reg_alpha	1.79368474695881	retune
MASLD	history	lightgbm	primary	reg_lambda	0.14666058476188978	retune
MASLD	history	lightgbm	primary	min_split_gain	0.033665087367061836	retune
MASLD	history	lightgbm	primary	subsample_freq	1	retune
MASLD	history	elasticnet	interpretable_secondary	C	0.08311381385109959	retune
MASLD	history	elasticnet	interpretable_secondary	l1_ratio	0.6605432156144846	retune

Supplementary Table 6B. Fold-specific tuning distributions underlying OOF performance

The minimum, median, and maximum values summarize the 50 independently tuned outer fits generated from 10 repeats of five outer folds for each specified configuration. Each outer-test prediction was produced using the parameter set selected within its corresponding outer-training data.

Outcome	Feature set	Model	Parameter	Outer fits	Minimum	Median	Maximum
HTN	history	lightgbm	n_estimators	50	91	239	449
HTN	history	lightgbm	learning_rate	50	0.010094	0.018054	0.086317
HTN	history	lightgbm	num_leaves	50	5	16	25
HTN	history	lightgbm	max_depth	50	2	3.5	6
HTN	history	lightgbm	min_child_samples	50	25	52.5	120
HTN	history	lightgbm	subsample	50	0.65045	0.80683	0.99936
HTN	history	lightgbm	colsample_bytree	50	0.55168	0.72245	0.99788
HTN	history	lightgbm	reg_alpha	50	1.0919e-05	0.0032985	4.0816
HTN	history	lightgbm	reg_lambda	50	1.0932e-05	0.037844	47.854
HTN	history	lightgbm	min_split_gain	50	0.003823	0.47455	0.99654
HTN	history	lightgbm	subsample_freq	50	1	1	1
HTN	history	elasticnet	C	50	0.025226	0.10014	0.27666
HTN	history	elasticnet	l1_ratio	50	0.047153	0.56205	0.99408
CHD	current	lightgbm	n_estimators	50	60	174	450
CHD	current	lightgbm	learning_rate	50	0.010371	0.018674	0.090801
CHD	current	lightgbm	num_leaves	50	5	16	25
CHD	current	lightgbm	max_depth	50	2	3	6
CHD	current	lightgbm	min_child_samples	50	25	70	113
CHD	current	lightgbm	subsample	50	0.65267	0.85345	0.99932
CHD	current	lightgbm	colsample_bytree	50	0.55026	0.74793	0.99946
CHD	current	lightgbm	reg_alpha	50	1.1352e-05	0.0096483	8.2077
CHD	current	lightgbm	reg_lambda	50	1.0298e-05	0.0035989	49.933
CHD	current	lightgbm	min_split_gain	50	0.0085088	0.57194	0.9954
CHD	current	lightgbm	subsample_freq	50	1	1	1
CHD	current	elasticnet	C	50	0.04753	0.10524	0.26744
CHD	current	elasticnet	l1_ratio	50	0.095878	0.56678	0.99535
DKD	baseline	elasticnet	C	50	0.19703	0.50876	18.91
DKD	baseline	elasticnet	l1_ratio	50	0.00015447	0.62035	0.99861
DR	current	elasticnet	C	50	0.059497	0.17105	0.39234
DR	current	elasticnet	l1_ratio	50	0.25441	0.72058	0.99591
MASLD	history	lightgbm	n_estimators	50	72	239.5	439
MASLD	history	lightgbm	learning_rate	50	0.010173	0.032074	0.10093
MASLD	history	lightgbm	num_leaves	50	5	15	25
MASLD	history	lightgbm	max_depth	50	2	4	6
MASLD	history	lightgbm	min_child_samples	50	27	81	117
MASLD	history	lightgbm	subsample	50	0.65088	0.74447	0.99512
MASLD	history	lightgbm	colsample_bytree	50	0.55411	0.81897	0.99938
MASLD	history	lightgbm	reg_alpha	50	1.4471e-05	0.027215	4.325
MASLD	history	lightgbm	reg_lambda	50	1.0628e-05	0.012641	46.86
MASLD	history	lightgbm	min_split_gain	50	0.0067244	0.543	0.99824
MASLD	history	lightgbm	subsample_freq	50	1	1	1
MASLD	history	elasticnet	C	50	0.063912	0.19295	0.53759
MASLD	history	elasticnet	l1_ratio	50	0.064263	0.8006	0.99964

Supplementary Table 6C. Model complexity and interpretation-stability summary

Candidate-feature counts are reported before fold-specific preprocessing and regularization. They describe the starting candidate dimensions rather than effective degrees of freedom. Because pooled rows contain repeated landmark records from the same individuals, the numbers of unique patients and events provide the key sample-size context. The valid refits

listed in this table refer to interpretation-stability analyses. Supplementary Table 7 summarizes model selection; Tables 8–9 summarize the 500-replicate model-development bootstrap.

Outcome	Feature set	Model	Role	Pooled rows	Patients	Events	Candidate features	Hash match	Valid bootstraps
HTN	history	lightgbm	primary	922	502	125	73	True	100
HTN	history	elasticnet	interpretable_secondary	922	502	125	73	True	100
CHD	current	lightgbm	primary	1145	634	201	40	True	100
CHD	current	elasticnet	interpretable_secondary	1145	634	201	40	True	100
DKD	baseline	elasticnet	primary	1203	637	113	7	True	100
DR	current	elasticnet	primary	1248	641	100	42	True	100
MASLD	history	lightgbm	primary	687	376	80	78	True	100
MASLD	history	elasticnet	interpretable_secondary	687	376	80	78	True	100

Supplementary Table 7. Fully nested adaptive model-selection sensitivity

Outcome	Selection distribution across 50 outer fits	Pooled AUC, mean (SD)	Pooled AP, mean (SD)	Pooled Brier, mean (SD)	Pooled CITL, mean (SD)	Pooled calibration slope, mean (SD)
HTN	history-LightGBM 47/50; history-elastic-net 3/50	0.786 (0.010)	0.384 (0.018)	0.100 (0.002)	-0.020 (0.029)	1.037 (0.096)
CHD	baseline-LightGBM 28/50; current-LightGBM 15/50; history-LightGBM 4/50; history-elastic-net 2/50; current-elastic-net 1/50	0.715 (0.007)	0.321 (0.010)	0.132 (0.001)	-0.004 (0.011)	0.987 (0.043)
DKD	baseline-elastic-net 33/50; baseline-LightGBM 13/50; current-elastic-net 4/50	0.755 (0.013)	0.276 (0.023)	0.077 (0.001)	-0.002 (0.015)	0.948 (0.083)
DR	current-elastic-net 37/50; history-elastic-net 10/50; history-LightGBM 2/50; current-LightGBM 1/50	0.919 (0.003)	0.556 (0.017)	0.051 (0.001)	-0.016 (0.047)	0.925 (0.029)
MASLD	history-LightGBM 49/50; history-elastic-net 1/50	0.950 (0.004)	0.729 (0.023)	0.054 (0.002)	-0.002 (0.050)	0.923 (0.052)

Across 50 outer fits per outcome, Supplementary Table 7 reports the complete nonzero selection distribution and pooled L1-L2 OOF performance of the adaptive selection procedure. The most frequently selected configuration matched the representative configuration for four of five outcomes, while CHD showed a broader selection distribution. Abbreviations: AP, average precision; AUC, area under the receiver operating characteristic curve; CITL, calibration-in-the-large; SD, standard deviation.

Supplementary Table 8. Formal 500-replicate model-development stability

Outcome	Representative configuration	Valid replicates	OOB AUC, median (2.5th-97.5th)	OOB AP, median (2.5th-97.5th)	OOB Brier, median (2.5th-97.5th)	OOB calibration slope, median (2.5th-97.5th)	Mean absolute prediction difference	Median development-instability width
HTN	history + LightGBM	500	0.775 (0.710-0.832)	0.382 (0.285-0.489)	0.103 (0.083-0.122)	1.068 (0.606-2.033)	0.057	0.151
CHD	current + LightGBM	500	0.713 (0.664-0.763)	0.333 (0.261-0.421)	0.133 (0.117-0.154)	1.097 (0.604-2.246)	0.045	0.167
DKD	baseline + elastic-net	500	0.763 (0.701-0.824)	0.312 (0.212-0.423)	0.076 (0.060-0.094)	1.038 (0.601-1.862)	0.017	0.054
DR	current + elastic-net	500	0.919 (0.882-0.946)	0.558 (0.439-0.668)	0.051 (0.040-0.064)	0.949 (0.688-1.402)	0.021	0.032
MASLD	history + LightGBM	500	0.952 (0.911-0.975)	0.745 (0.600-0.868)	0.055 (0.038-0.077)	1.012 (0.674-1.574)	0.036	0.028

Out-of-bag metrics summarize performance in patients not sampled into each bootstrap development set, while prediction-instability measures summarize variation in original-cohort predictions across bootstrap-developed models. All 500 bootstrap replicates yielded evaluable pooled OOB results for all five representative configurations. The reported ranges characterize model-development variability and are not individual-level risk intervals.

Supplementary Table 9. Pooled L1-L2 paired out-of-bag bootstrap contrasts

Outcome	Contrast	Configuration	Valid paired replicates	ΔAUC median (2.5th-97.5th)	AUC favorable direction	ΔAP median (2.5th-97.5th)	ΔBrier median (2.5th-97.5th)
HTN	history-current	elastic-net	500	0.091 (0.036, 0.297)	0.994	0.063 (-0.018, 0.211)	-0.006 (-0.015, 0.003)
HTN	history-current	LightGBM	500	0.117 (0.047, 0.190)	1.000	0.126 (0.018, 0.227)	-0.011 (-0.019, -0.003)
HTN	LightGBM-elastic-net	current	500	-0.025 (-0.088, 0.149)	0.216	-0.015 (-0.107, 0.115)	0.001 (-0.006, 0.006)
HTN	LightGBM-elastic-net	history	500	-0.002 (-0.053, 0.047)	0.456	0.043 (-0.039, 0.136)	-0.003 (-0.012, 0.005)
CHD	history-current	elastic-net	500	-0.007 (-0.060, 0.025)	0.340	-0.011 (-0.089, 0.038)	0.001 (-0.004, 0.008)
CHD	history-current	LightGBM	500	-0.003 (-0.044, 0.030)	0.442	-0.006 (-0.073, 0.048)	0.000 (-0.005, 0.007)
CHD	LightGBM-elastic-net	current	500	0.022 (-0.021, 0.069)	0.816	-0.007 (-0.075, 0.071)	-0.001 (-0.008, 0.006)
CHD	LightGBM-elastic-net	history	500	0.026 (-0.020, 0.081)	0.850	0.004 (-0.067, 0.071)	-0.002 (-0.009, 0.006)
DKD	history-current	elastic-net	500	-0.008 (-0.056, 0.025)	0.302	-0.014 (-0.090, 0.047)	0.001 (-0.003, 0.006)
DKD	history-current	LightGBM	500	-0.004 (-0.052, 0.034)	0.422	0.002 (-0.067, 0.070)	0.000 (-0.003, 0.004)

Outcome	Contrast	Configuration	Valid paired replicates	ΔAUC median (2.5th-97.5th)	AUC favorable direction	ΔAP median (2.5th-97.5th)	ΔBrier median (2.5th-97.5th)
DKD	LightGBM-elastic-net	current	500	-0.021 (-0.072, 0.032)	0.218	-0.023 (-0.108, 0.065)	0.001 (-0.004, 0.006)
DKD	LightGBM-elastic-net	history	500	-0.017 (-0.070, 0.046)	0.270	-0.006 (-0.102, 0.079)	0.001 (-0.005, 0.005)
DR	history-current	elastic-net	500	-0.001 (-0.030, 0.017)	0.452	-0.011 (-0.105, 0.051)	0.001 (-0.004, 0.007)
DR	history-current	LightGBM	500	-0.001 (-0.023, 0.021)	0.452	0.003 (-0.078, 0.065)	-0.000 (-0.005, 0.004)
DR	LightGBM-elastic-net	current	500	-0.004 (-0.030, 0.020)	0.366	-0.037 (-0.122, 0.040)	0.002 (-0.003, 0.008)
DR	LightGBM-elastic-net	history	500	-0.003 (-0.029, 0.027)	0.424	-0.023 (-0.112, 0.070)	0.001 (-0.005, 0.007)
MASLD	history-current	elastic-net	500	0.019 (-0.023, 0.055)	0.832	0.042 (-0.059, 0.136)	-0.004 (-0.015, 0.008)
MASLD	history-current	LightGBM	500	0.025 (-0.002, 0.060)	0.960	0.063 (-0.044, 0.154)	-0.007 (-0.017, 0.006)
MASLD	LightGBM-elastic-net	current	500	0.017 (-0.020, 0.067)	0.784	0.044 (-0.073, 0.161)	-0.003 (-0.017, 0.008)
MASLD	LightGBM-elastic-net	history	500	0.023 (-0.011, 0.071)	0.886	0.060 (-0.065, 0.184)	-0.007 (-0.022, 0.009)

Supplementary Table 9 reports the median difference, 2.5th-97.5th percentile development-stability range, favorable-direction proportion, and number of valid paired replicates for the prespecified history-current and LightGBM-elastic-net comparisons. For ΔAUC and ΔAP, a positive value favors history or LightGBM as named in the contrast. For ΔBrier, a negative value is favorable.

2.3 Supplementary Table 10. Operational outcome coding and transition audit

Outcome	Status 0	Status 1	Missing or other	Patients with 1-to-0	Cells changed by carry-forward
HTN	1945	639	0	0	0
CHD	2225	359	0	1	2
DKD	2373	211	0	0	0
DR	2435	149	0	0	0
MASLD	1640	944	0	8	11

Note. Counts for status 0 and status 1 are across all 2,584 source T0-T3 annual outcome-status records before carry-forward. Status 0 denotes that the target outcome was not recorded as present at that annual assessment; status 1 denotes that it was recorded as present. The final extract contained no missing outcome status or separate uncertain category after the two-investigator eligibility, consistency, and coding review. Carry-forward was applied outcome by outcome only in the primary analytic coding after the first status 1; the counts in this table describe the source annual fields before that rule.

2.4 Supplementary Table 11. L1 T3-masking data-flow audit

Outcome	L1 at-risk patients	L1 events	Unchanged feature sets	Overall status
HTN	502	82	4/4	Passed
CHD	634	123	4/4	Passed
DKD	637	71	4/4	Passed
DR	641	34	4/4	Passed
MASLD	376	65	4/4	Passed

Note. All T3 dynamic values were deliberately altered before reconstruction of the L1 landmark-only, baseline, current, and history datasets. Passing required identical patient membership, event labels, predictor columns, and predictor values. This is a temporal data-use audit within the fixed 646-patient cohort. It does not address selection caused by the complete-follow-up eligibility restriction.

2.5 Supplementary Figures

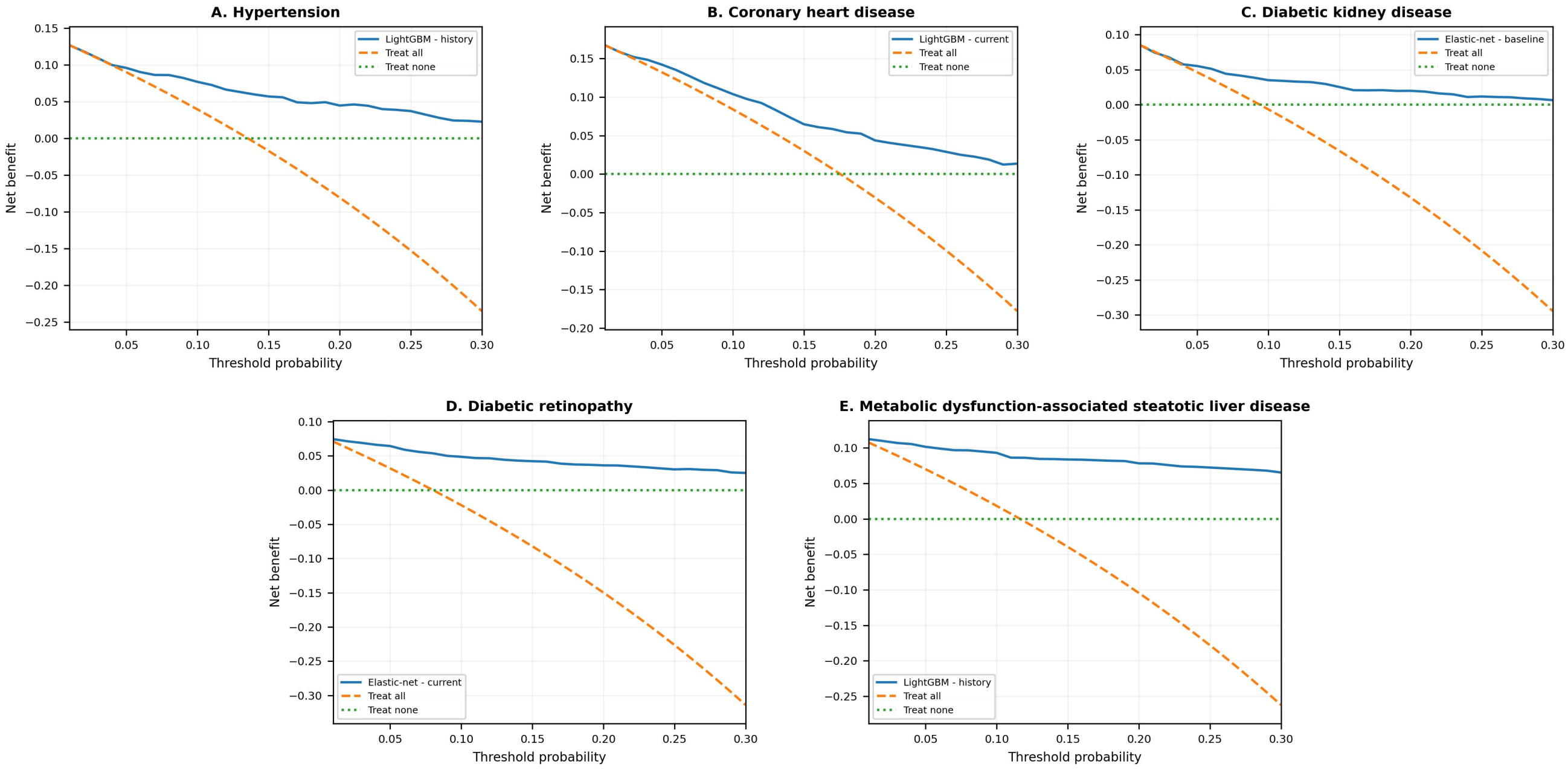

Supplementary Figure 1. Exploratory decision-curve analysis for the five representative models. Panels A-E show pooled L1-L2 decision curves for the HTN history-LightGBM, CHD current-LightGBM, DKD baseline-elastic-net, DR current-elastic-net, and MASLD history-LightGBM configurations, respectively. Model curves were derived from repeated-CV OOF probabilities after averaging the 10 OOF probabilities for each patient-landmark record. Treat-all and treat-none strategies are shown for reference. Net benefit was evaluated over threshold probabilities from 0.01 to 0.50, while the figure displays 0.01 to 0.30 for greater visual resolution. Representative-model selection was completed independently of this supplementary analysis.

Supplementary Figure 2. Individual prediction stability across 500 bootstrap-developed models

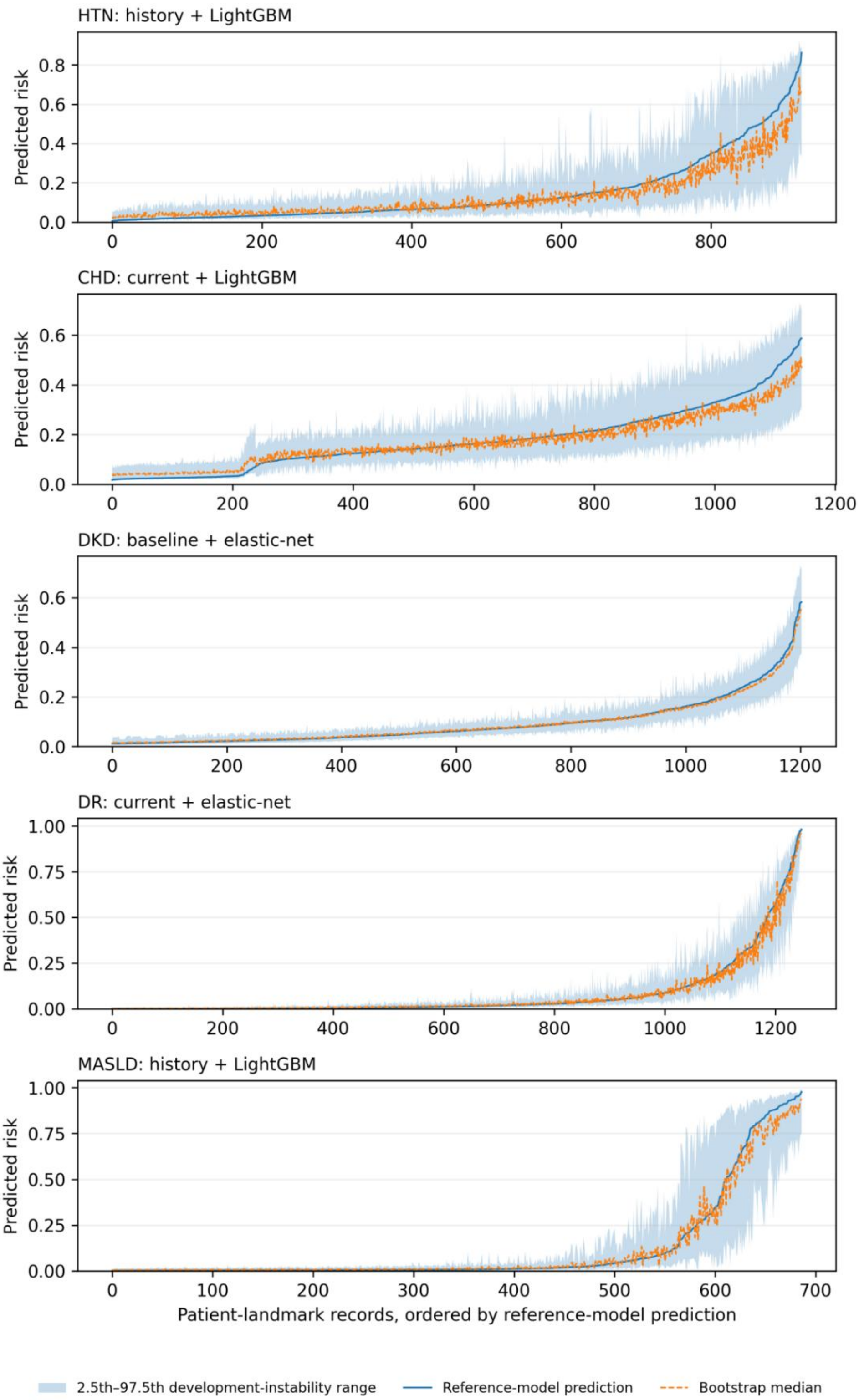

Supplementary Figure 2. Individual prediction stability across 500 bootstrap-developed models. Within each representative configuration, patient-landmark records are ordered by the reference-model predicted risk. The solid blue line shows the reference-model prediction, the dashed orange line shows the median prediction across bootstrap-developed models, and the shaded region shows the 2.5th-97.5th percentile development-instability range. The shaded range characterizes model-development stability and is not an individual-risk confidence interval.

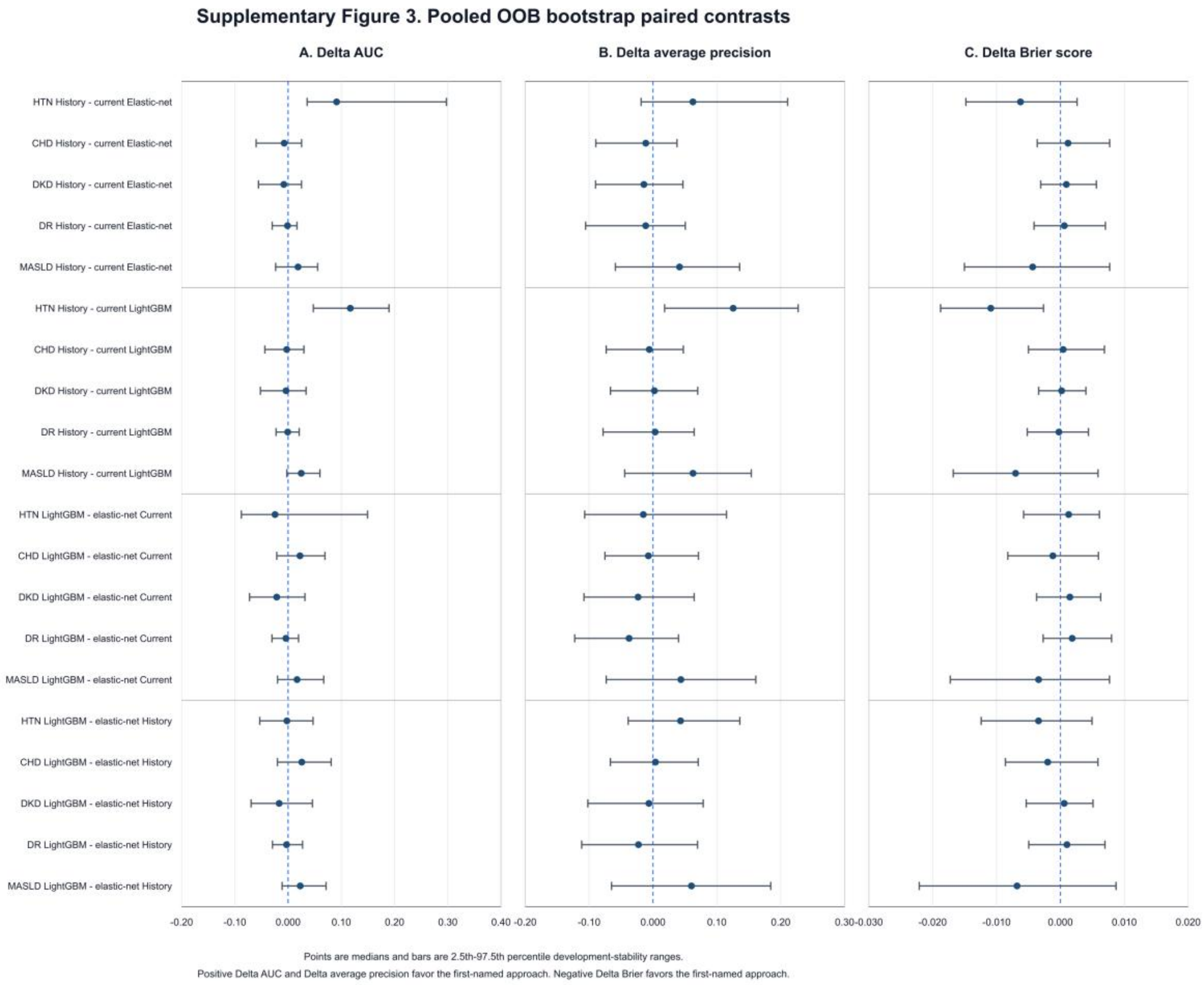

Supplementary Figure 3. Pooled out-of-bag bootstrap distributions of the prespecified paired contrasts. Points show the median difference and horizontal bars show the 2.5th-97.5th percentile development-stability range across 500 patient-level bootstrap replicates. Contrasts compare history with current features within each algorithm and LightGBM with elastic-net within the current and history feature sets. Positive ΔAUC and ΔAP values favor the first-named approach, whereas negative ΔBrier values favor the first-named approach.

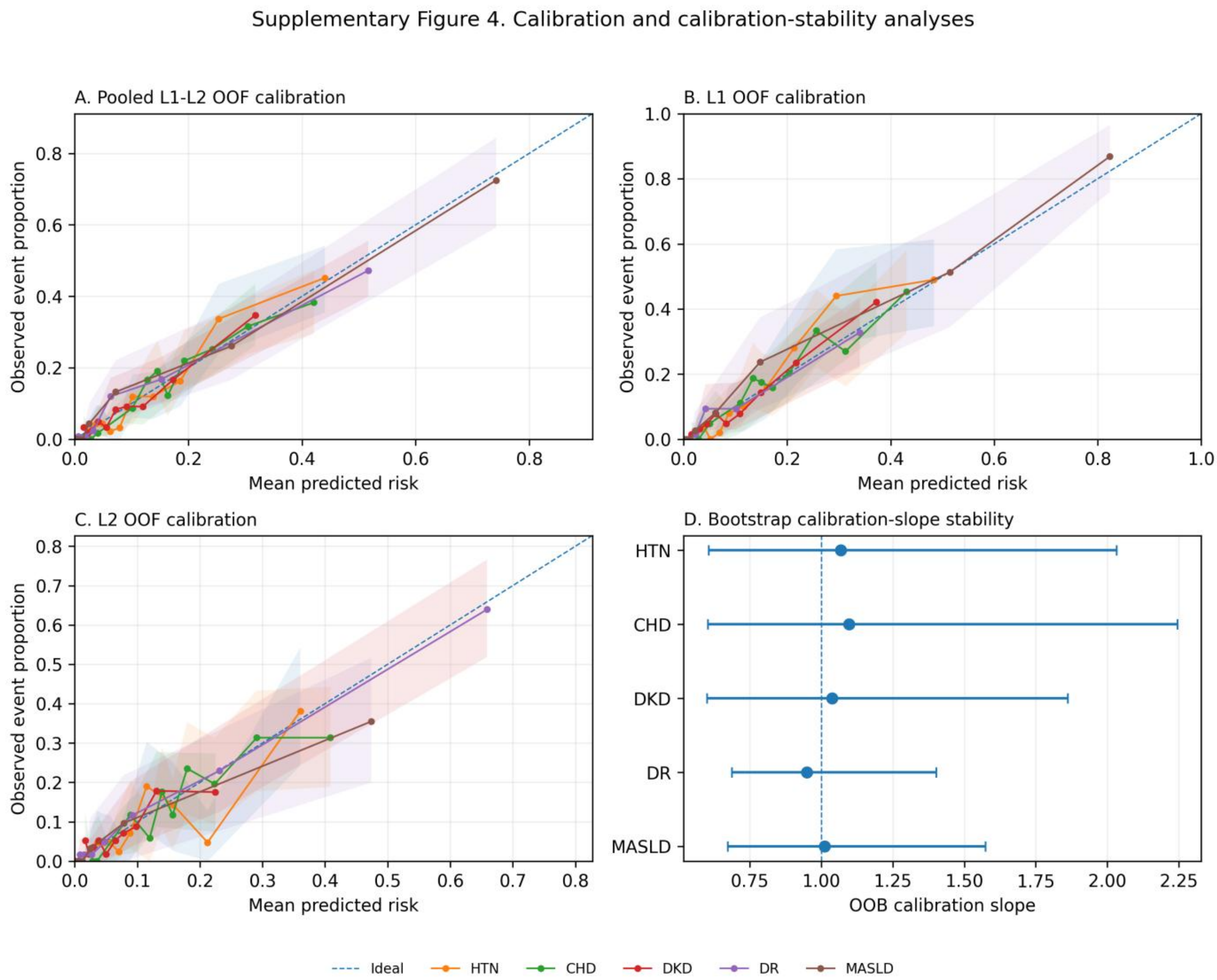

Supplementary Figure 4. Calibration and calibration-stability analyses. Panels A-C show repeated-CV out-of-fold calibration curves with patient-level bootstrap uncertainty bands for pooled L1-L2, L1, and L2 predictions. The dashed diagonal denotes ideal calibration. Panel D shows the median and 2.5th-97.5th percentile range of out-of-bag calibration slopes across the 500 model-development bootstrap replicates for the five representative configurations. The vertical dashed line in Panel D denotes a calibration slope of 1.
